# Supplementary material for: Highly durable crack sensor integrated with silicone rubber cantilever for measuring cardiac contractility
Source: Nat Commun. 2020 Jan 27;11:535. doi: 10.1038/s41467-019-14019-y (PMC6985253; doi:10.1038/s41467-019-14019-y)
Supplement: Supplementary file 1 — Supplementary Information [file 41467_2019_14019_MOESM1_ESM.pdf]

Supplementary Information
For

Highly durable crack sensor integrated with silicone rubber cantilever for measuring cardiac contractility

Kim et al.

Supplementary Figures

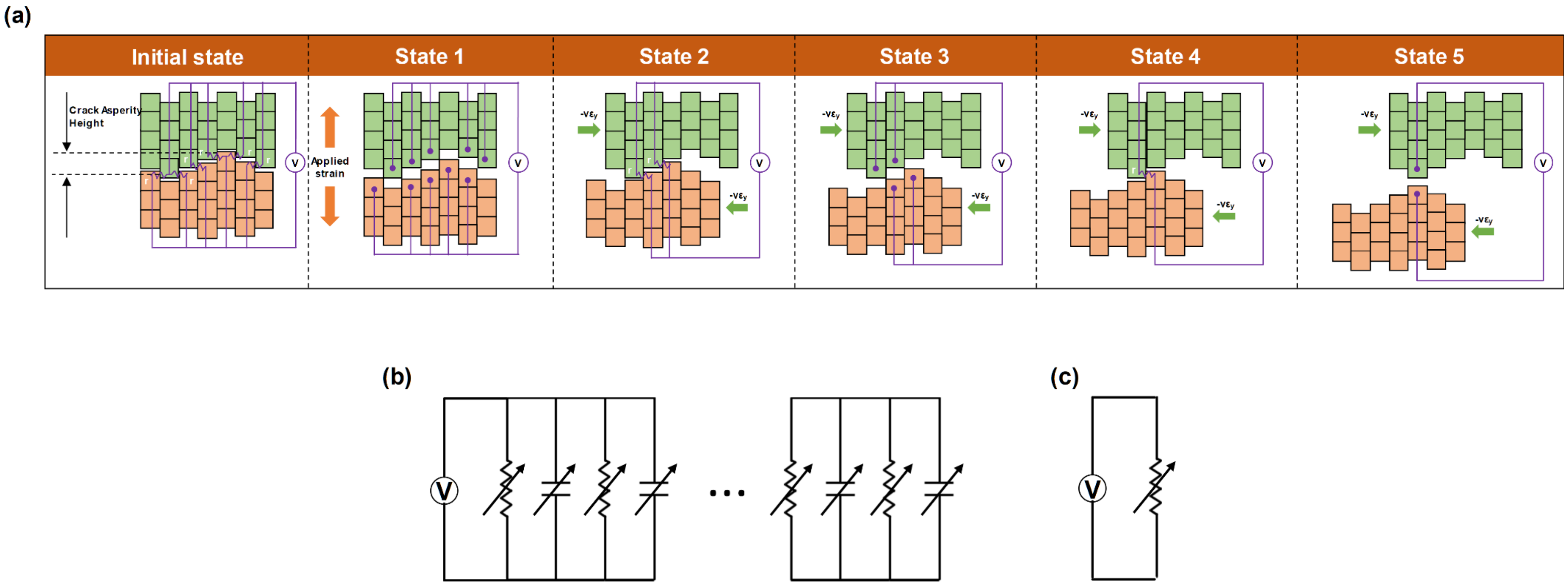

Supplementary Figure. 1. Conceptual diagram depicting the working principle of the crack sensors. **a**, Diagram illustrates the operating principle of the proposed crack sensor. **b**, **c**, Equivalent circuit modeling of the proposed PDMS-encapsulated crack sensor integrated silicone rubber cantilever.

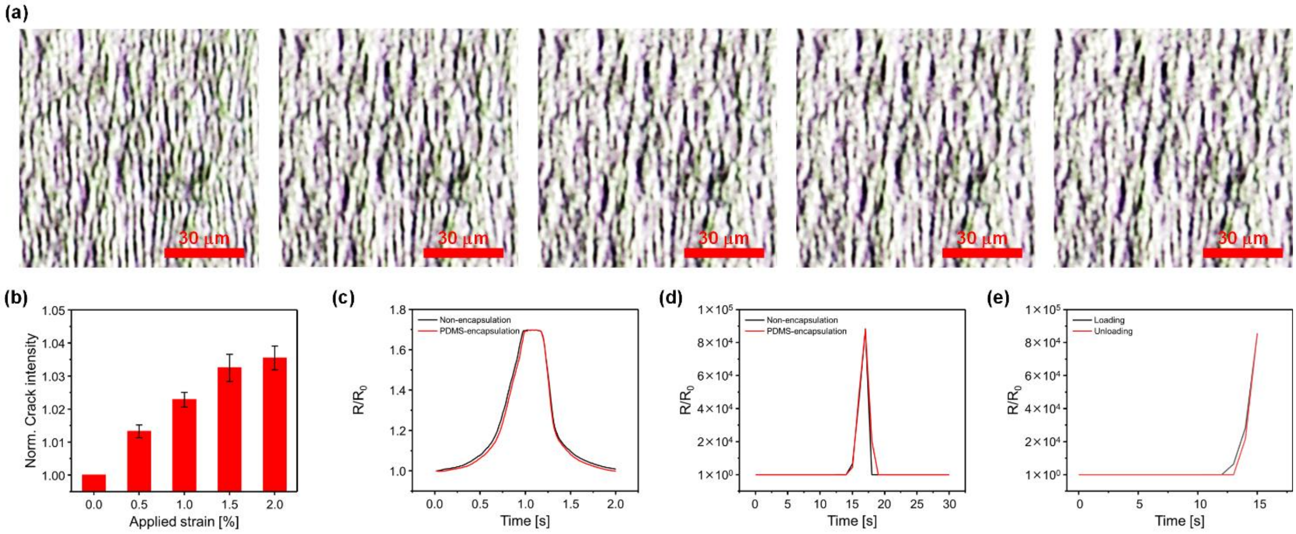

Supplementary Figure. 2. Effect of applied strain on the crack intensity and change in resistance ratio of the crack sensors. **a**, Optical microscope images of the PDMS-encapsulated crack sensor before and after stretching (ε = 0, 0.5, 1, 1.5, 2%). **b**, The intensity of cracks according to the applied tensile strain. **c**, Resistance changes as a function of strain ranging from 0 to 0.3%. **d**, Resistance changes as a function of strain ranging from 0 to 1%. **e**, Hysteresis of the PDMS-encapsulated crack sensor. Error bars are mean ± s.d. (n=5).

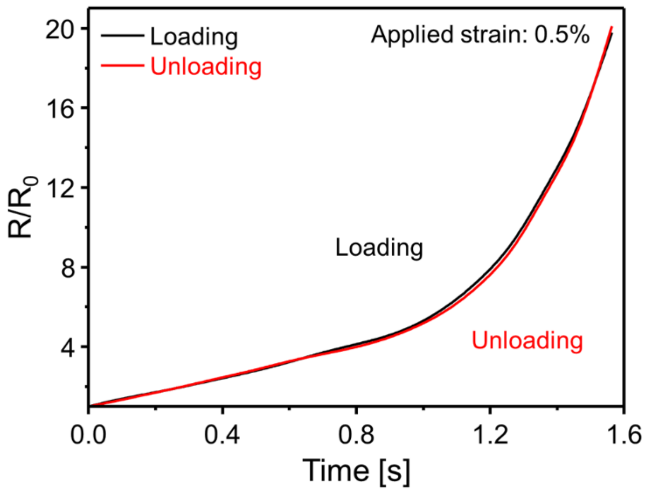

Supplementary Figure. 3. Hysteresis of the PDMS-encapsulated crack sensor integrated silicone rubber cantilever. Change in resistance ratio of the PDMS-encapsulated crack sensor integrated silicone rubber cantilever during loading and unloading of applied strain in the tensile range of 0-0.5%.

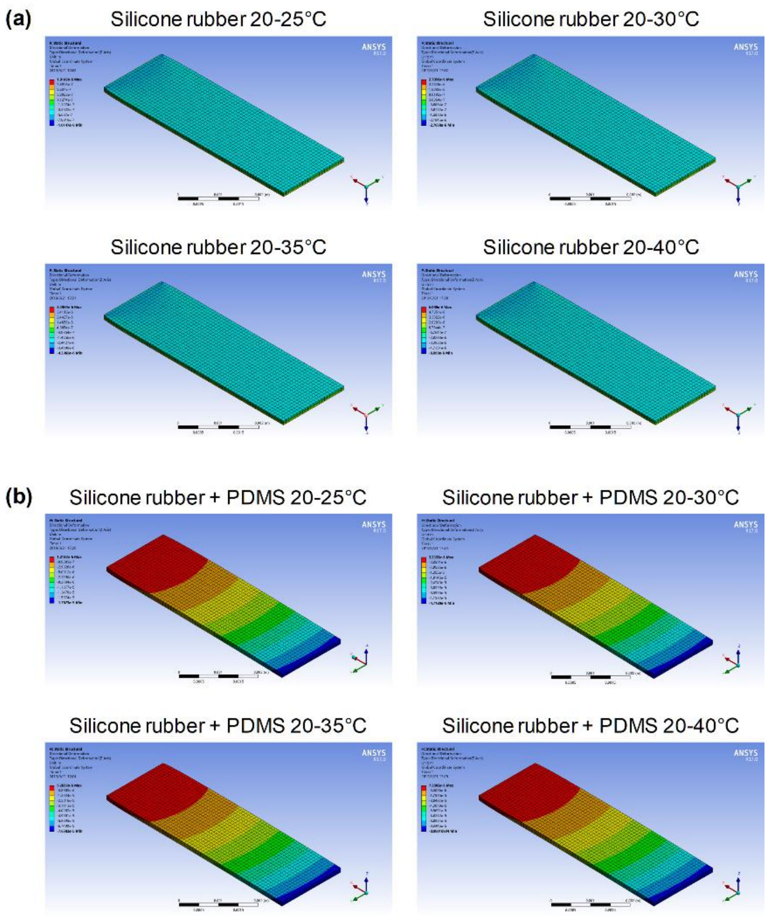

Supplementary Figure. 4. Finite-element method modeling results of silicone rubber and PDMS, silicone sandwich structures. **a**, **b**, Thermal-expansion analysis of cantilever made by the silicone rubber and PDMS + silicone rubber at different temperatures.

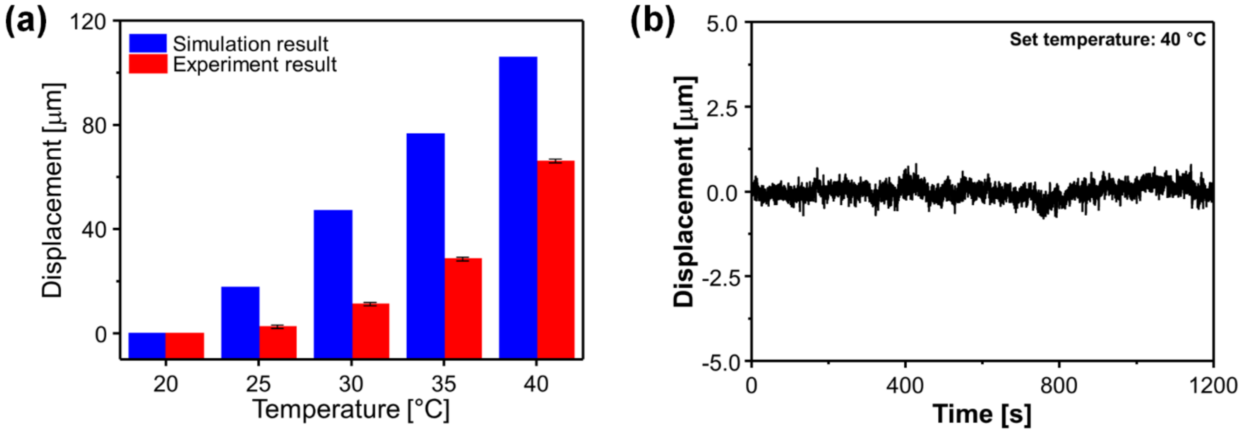

Supplementary Figure. 5. Experimental and theoretical displacement of the silicone rubber cantilever. **a**, Displacement of the silicone rubber cantilever at different temperatures. **b**, The displacement of the silicone rubber cantilever at a constant temperature environment (40 °C). Error bars are mean ± s.d. (n=5).

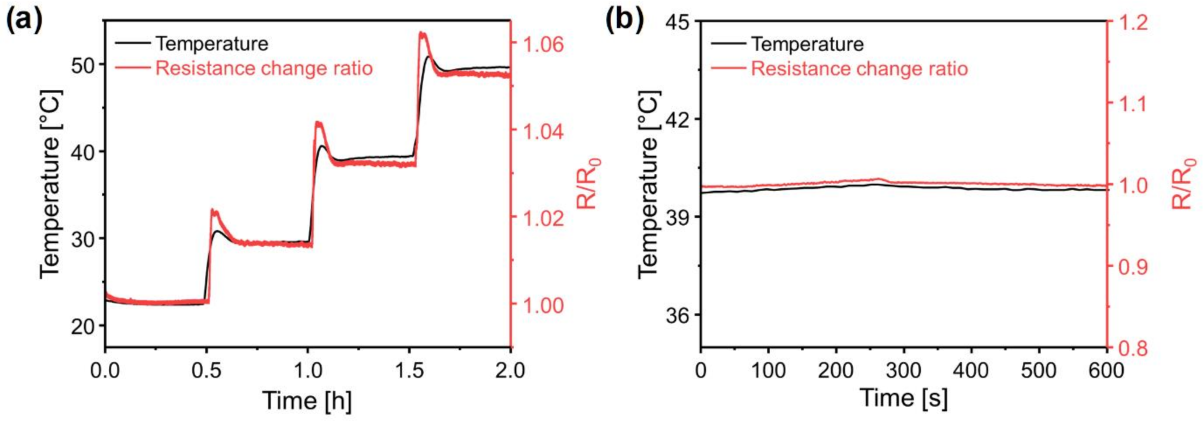

Supplementary Figure. 6. Effect of temperature on the resistance of the PDMS-encapsulated crack sensor. **a**, Change in resistance of the PDMS-encapsulated crack sensor integrated silicone rubber cantilever as a function of time at different temperatures. **b**, Change in resistance of the PDMS-encapsulated crack sensor integrated silicone rubber cantilever as a function of time under the home-made stage top incubator at 40 °C.

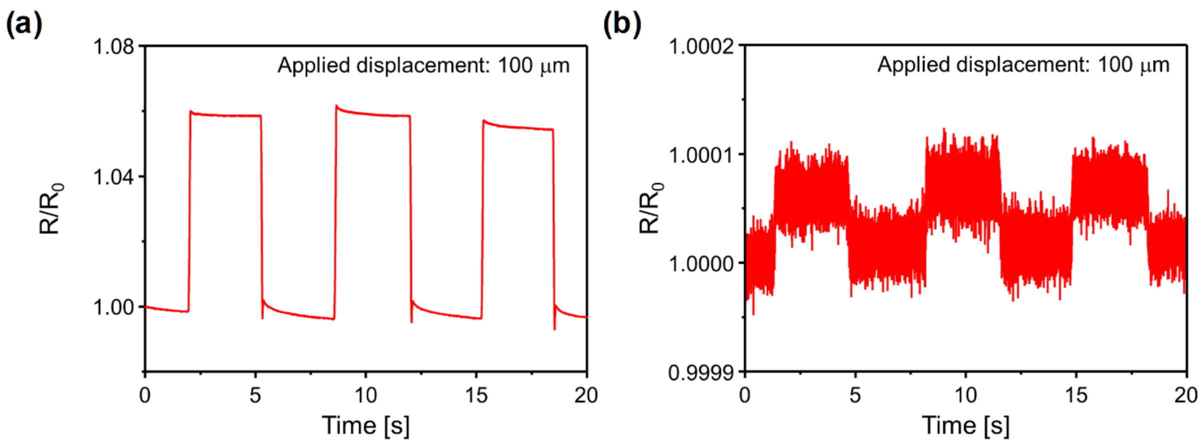

Supplementary Figure. 7. Real-time change in resistance ratio of the commercial strain sensor and PDMS-encapsulated crack sensor. **a**, **b**, Representative traces of real-time change in resistance of the proposed crack sensor and commercial Au strain sensor at 100 μm displacement.

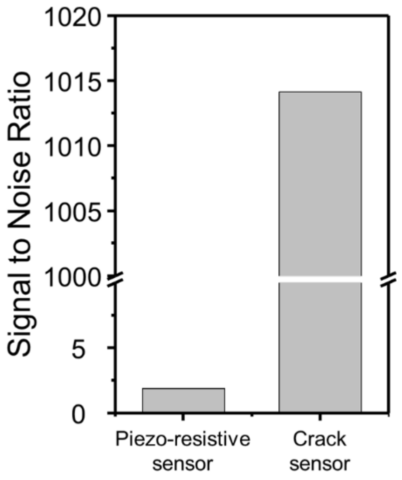

Supplementary Figure. 8. Signal to noise (SNR) of resistance change of the commercial strain sensor and PDMS-encapsulated crack sensor. Bar plot representing the SNR of resistance change of piezo-resistive sensor and PDMS-encapsulated crack sensor owing to the contraction force of the cultured cardiomyocytes.

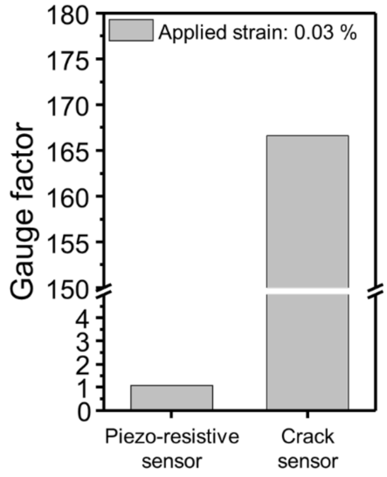

Supplementary Figure. 9. Gauge factor of the commercial strain sensor and PDMS-encapsulated crack sensor. Bar plot representing the gauge factor of the piezo-resistive sensor and PDMS-encapsulated crack sensor at 0.03% of applied strain.

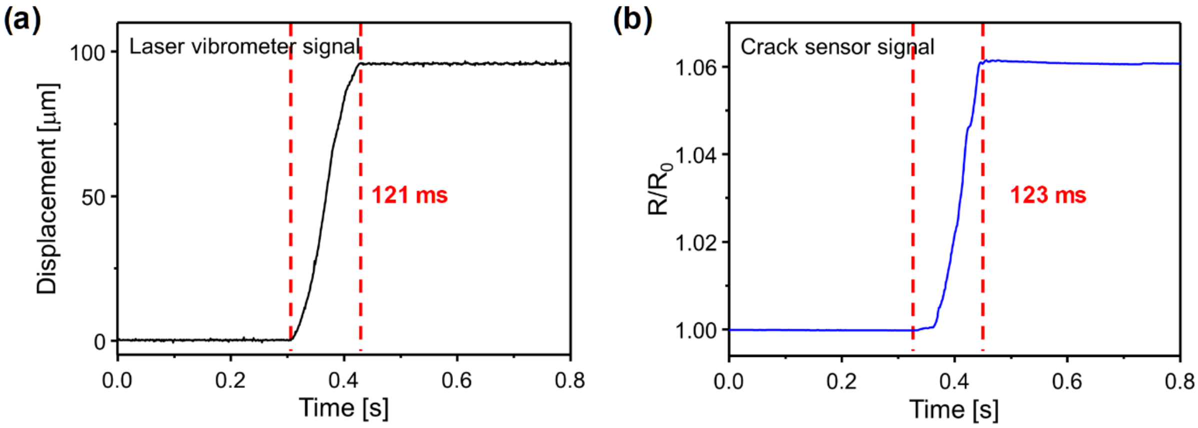

Supplementary Figure. 10. Response time of the cantilever. A comparison of the response time of the PDMS-encapsulated crack sensor integrated silicone rubber cantilever measured using **a**, laser vibrometer and **b**, integrated crack sensor.

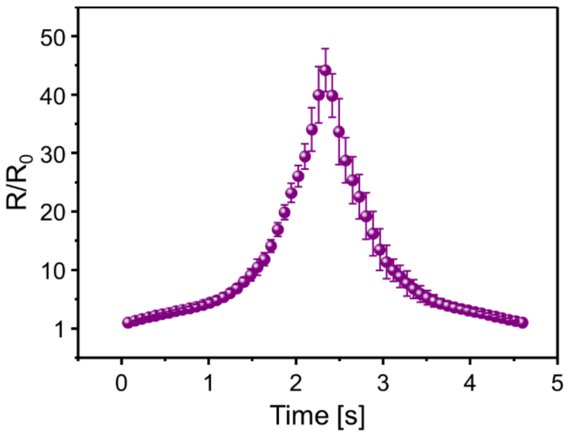

Supplementary Figure. 11. Electrical properties of the PDMS-encapsulated crack sensors. Change in resistance ratio and standard deviation of the five different PDMS-encapsulated crack sensors at the different applied strain in the range of 0 – 0.7%. Error bars are mean ± s.d. (n=5).

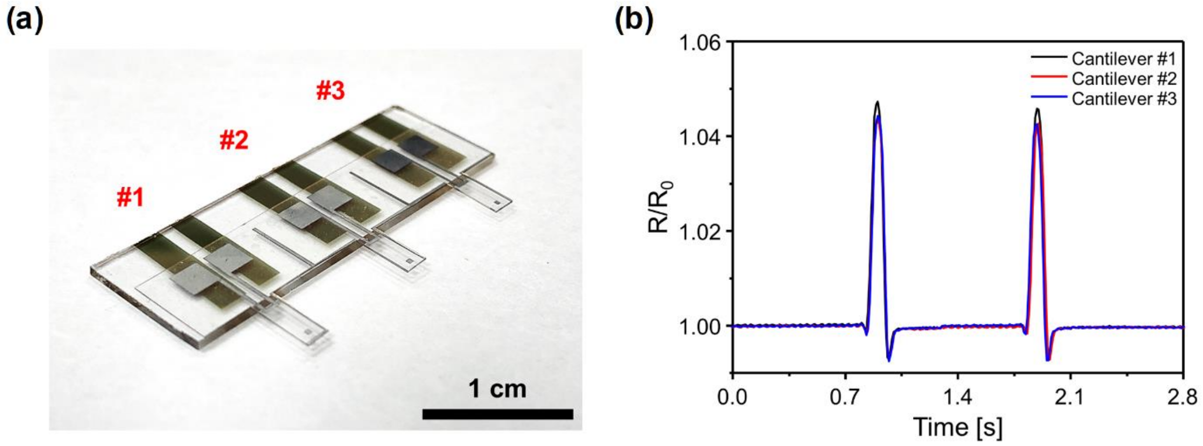

Supplementary Figure. 12. Repeatability and reproducibility of crack sensors. **a**, Optical image of the fabricated multi- PDMS-encapsulated crack sensor integrated with silicone rubber cantilever arrays. **b**, Change in resistance ratio of the multi-cantilever arrays as a function of applied strain in the range of 0 to 0.03%.

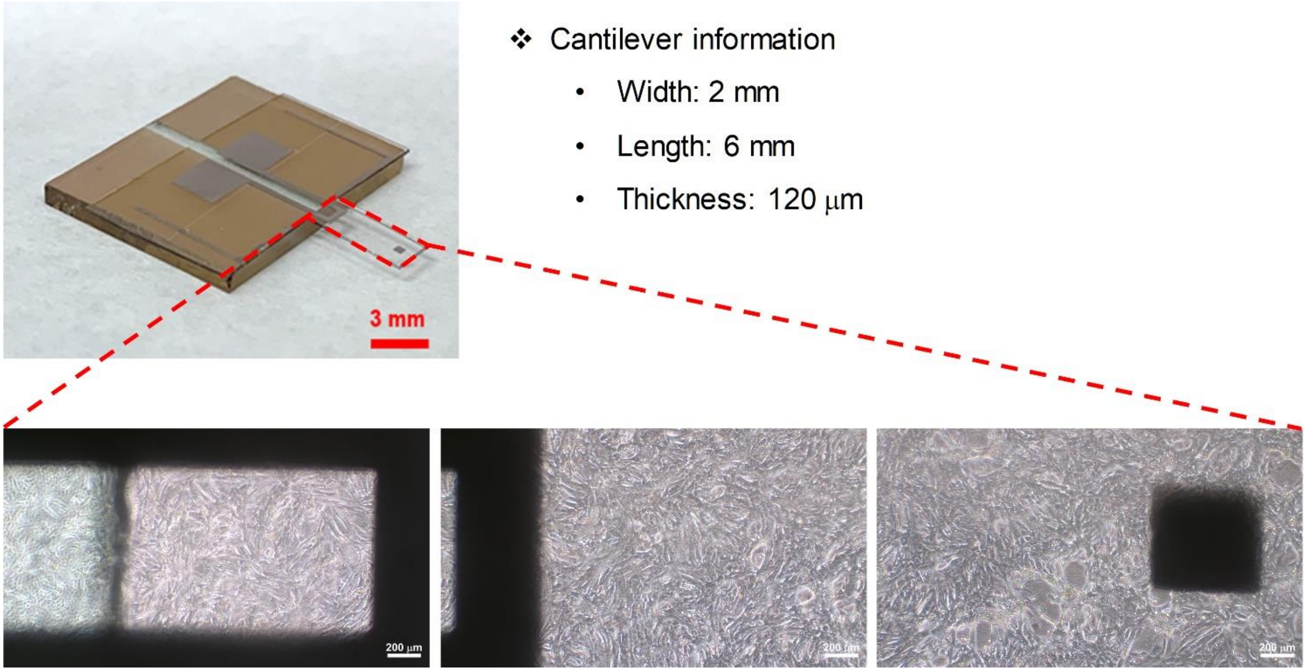

Supplementary Figure. 13. Morphology of the cardiomyocytes. Optical images of the silicone rubber cantilever integrated with the PDMS-encapsulated crack sensor and cardiac cells grown at different parts of the silicone rubber cantilever (Fixed, middle, and free ends of the cantilever).

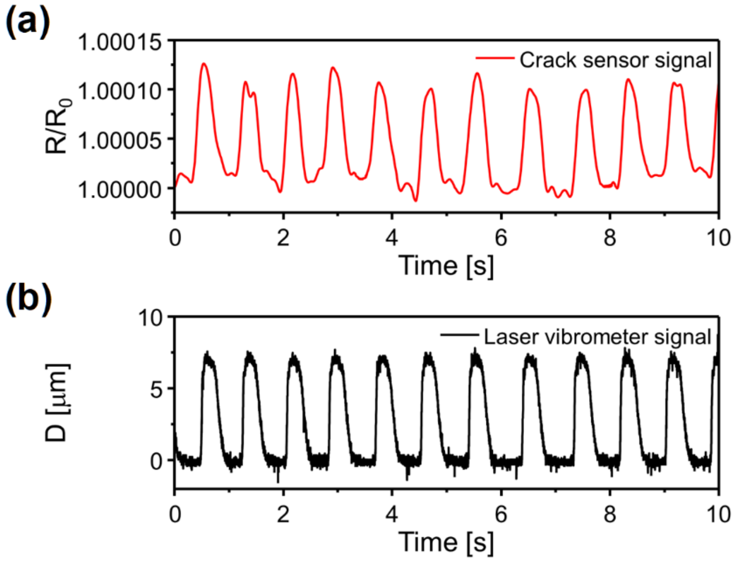

Supplementary Figure. 14. Electrical and mechanical properties of the cultured cardiomyocytes. **a**, Change in resistance ratio of the cardiomyocytes seeded PDMS-encapsulated crack sensor integrated with silicone rubber cantilever due to contraction and relaxation of cardiac cells. **b**, Displacement of the cardiomyocytes seeded PDMS-encapsulated crack sensor integrated with silicone rubber cantilever in the crack sensor due to the contraction and relaxation of cardiac cells.

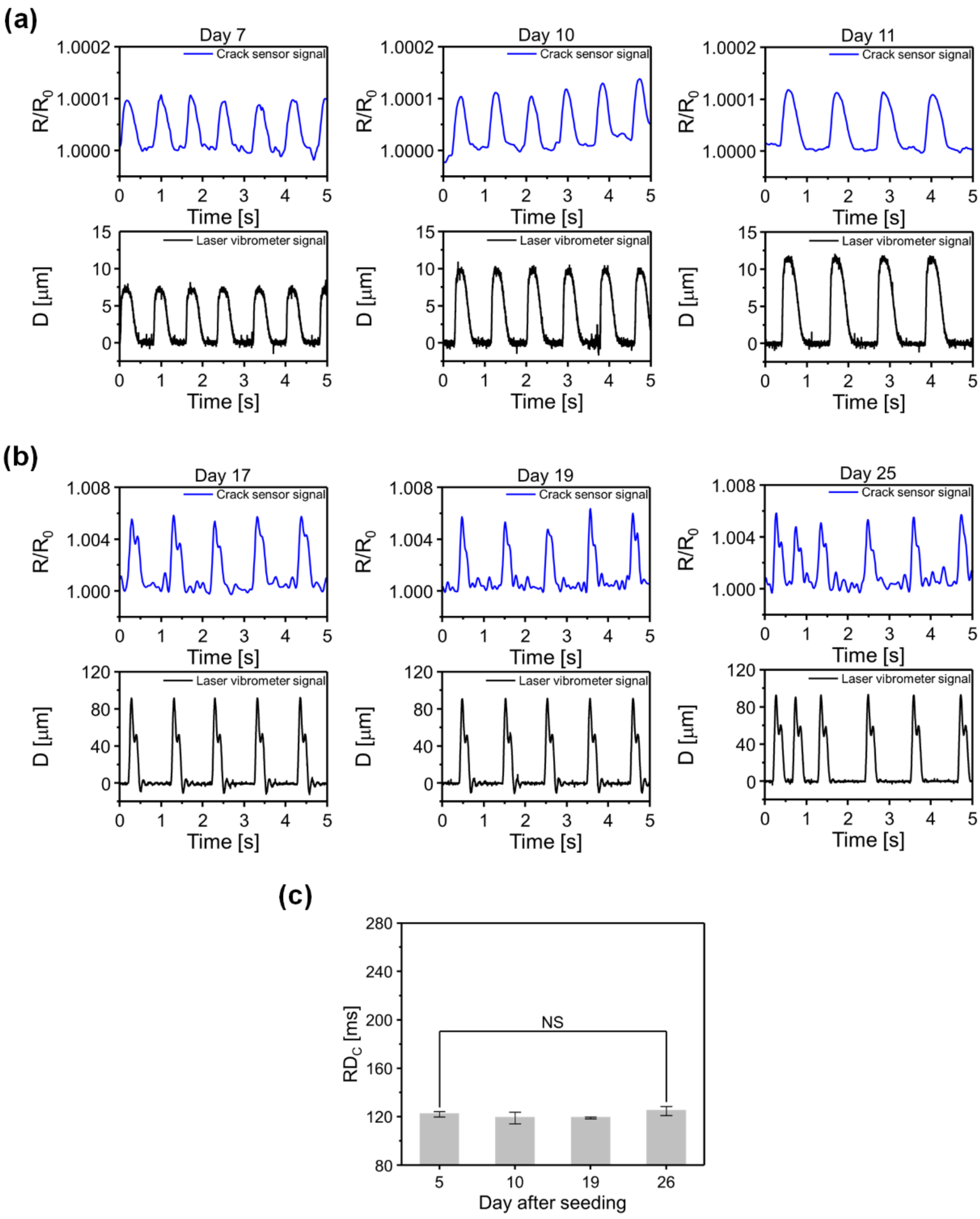

Supplementary Figure. 15. Long-term stability of the PDMS-encapsulated crack sensor integrated with silicone rubber cantilever. **a**, **b**, Representative traces of real-time change in resistance ratio and displacement of the cardiomyocytes cultured PDMS-encapsulated crack sensor at different culture days. Rise and decay time of cultured cardiomyocytes measured over 26 days. **c**, The rise and decay time (RD) for contractility analysis of the cultured cardiomyocytes were beat rate corrected (RDc) with Fridericia's formula (RDc = RD/interspike interval$^{1/3}$). (Non-significant (NS) and measures by two-way ANOVA followed by Tukey's honest significant difference test.). Error bars are mean ± s.d. (n=5 biologically independent samples). D is representing displacement.

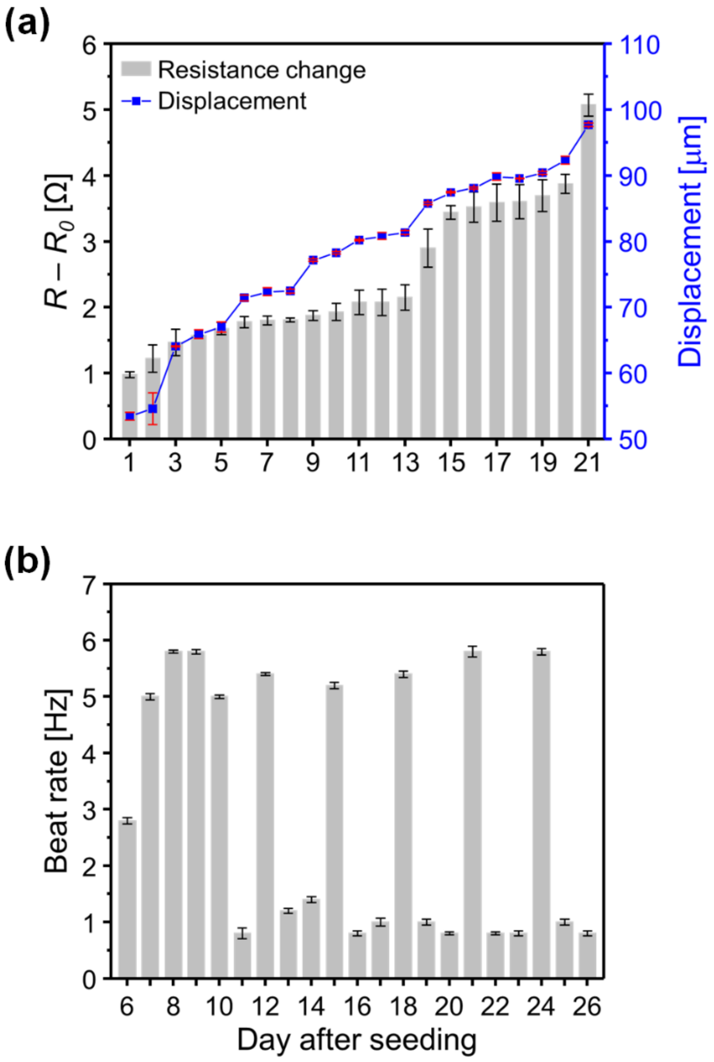

Supplementary Figure. 16. Electrical and mechanical properties of the cardiomyocytes cultured cantilever. **a**, Resistance changes and displacement of the PDMS-encapsulated crack sensor integrated with silicone rubber cantilever owing to the contractility of the cultured cardiomyocytes over 26 days. **b**, Beating frequency of the cultured cardiomyocytes as a function of culture days. Error bars are mean ± s.d. (n=5 biologically independent samples).

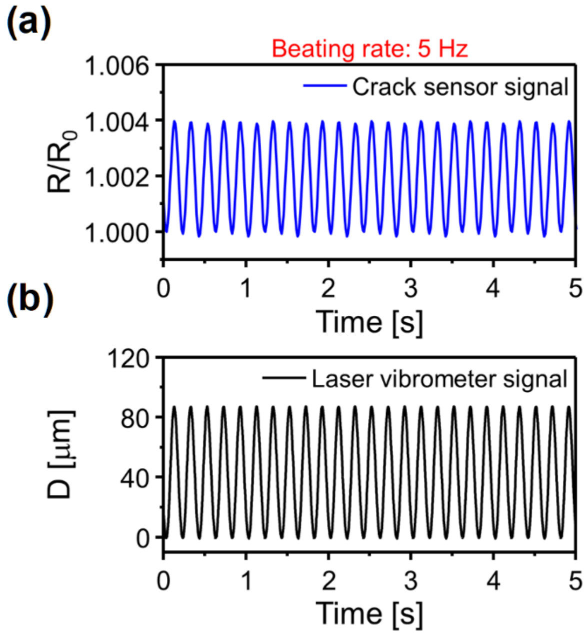

Supplementary Figure. 17. Response and recovery characteristics of the cantilever. **a**, Change in resistance ratio of the PDMS-encapsulated crack sensor integrated with silicone rubber cantilever owing to the contractility of the cultured cardiomyocytes. **b**, Displacement of the PDMS-encapsulated crack sensor integrated with silicone rubber cantilever due to the contractility of the cultured cardiomyocytes measured by the laser vibrometer.

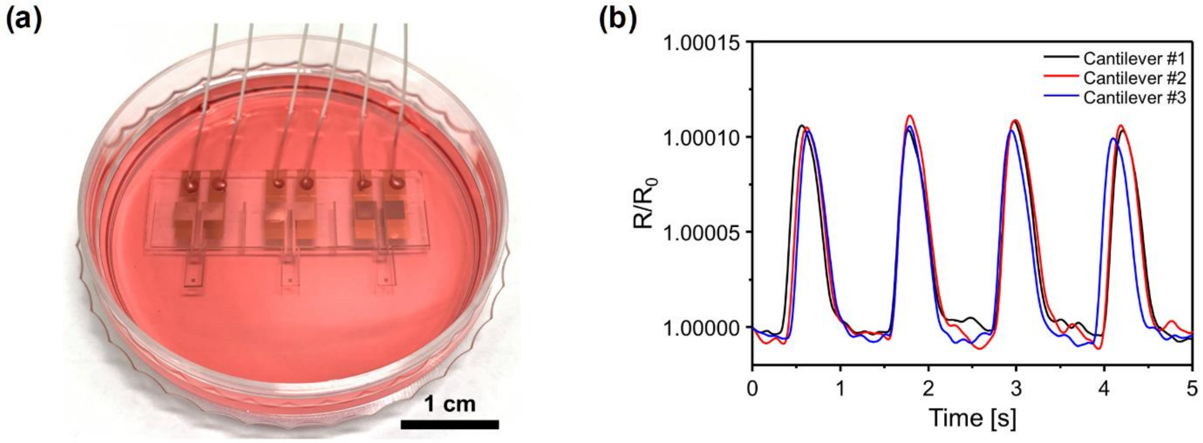

Supplementary Figure. 18. Repeatability and reproducibility of crack sensors under the cell culture medium. **a**, Photograph of a multi-cantilever device consisting of three PDMS-encapsulated crack sensors integrated with silicone rubber cantilever in a cell culture medium. **b**, Change in resistance ratio of the PDMS-encapsulated crack sensor integrated with silicone rubber cantilever owing to the contractile force of cultured cardiomyocytes.

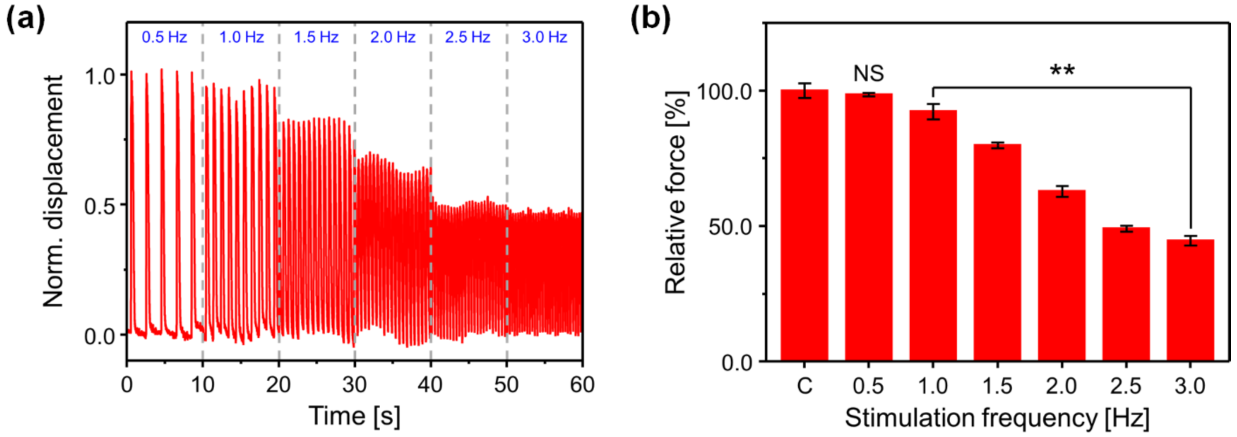

Supplementary Figure. 19. Effect of external electrical stimulation on the cultured cardiomyocytes. **a**, Force-frequency relationships of cultured cardiomyocytes as a function of time. **b**, Relative contraction force generated by cardiomyocytes at different applied electrical stimulation frequencies. Error bars are mean ± s.d. (n=5 biologically independent samples); ** $p < 0.01$ measures by one-way ANOVA followed by Tukey's honest significant difference test. C and NS are representing control and non-significant.

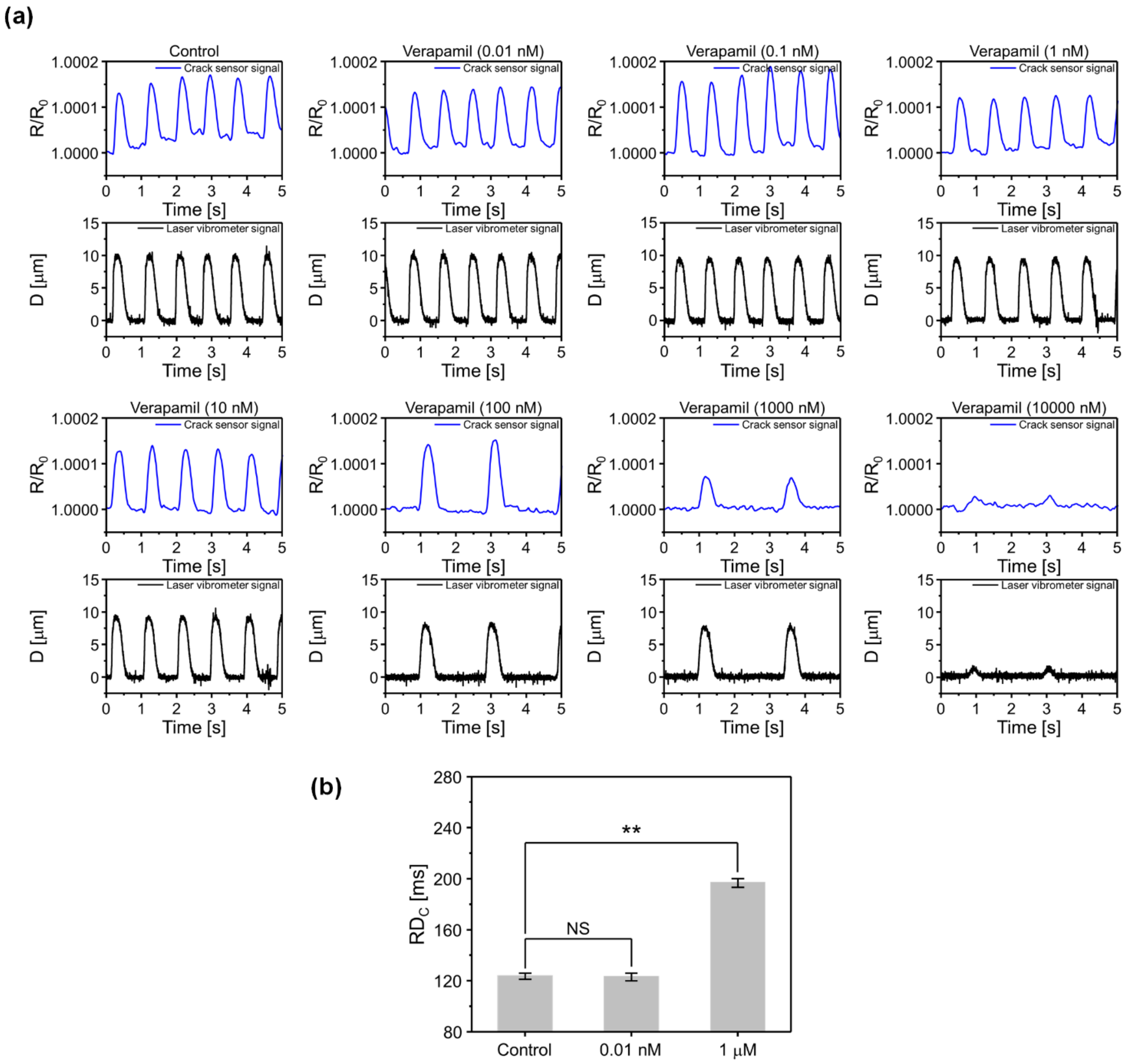

Supplementary Figure. 20. Real-time traces are representing the contractility of the cultured cardiomyocytes. **a**, Experimental results of cardiac contractile force and beating frequency according to Verapamil concentration. **b**, The rise and decay time (RD) for contractility analysis of the cultured cardiomyocytes were beat rate corrected (RDc) with Fridericia's formula (RDc = RD/interspike interval$^{1/3}$). (Non-significant (NS) and ** $p < 0.01$ measures by one-way ANOVA followed by Tukey's honest significant difference test.). Error bars are mean ± s.d. (n=5 biologically independent samples). D is representing displacement.

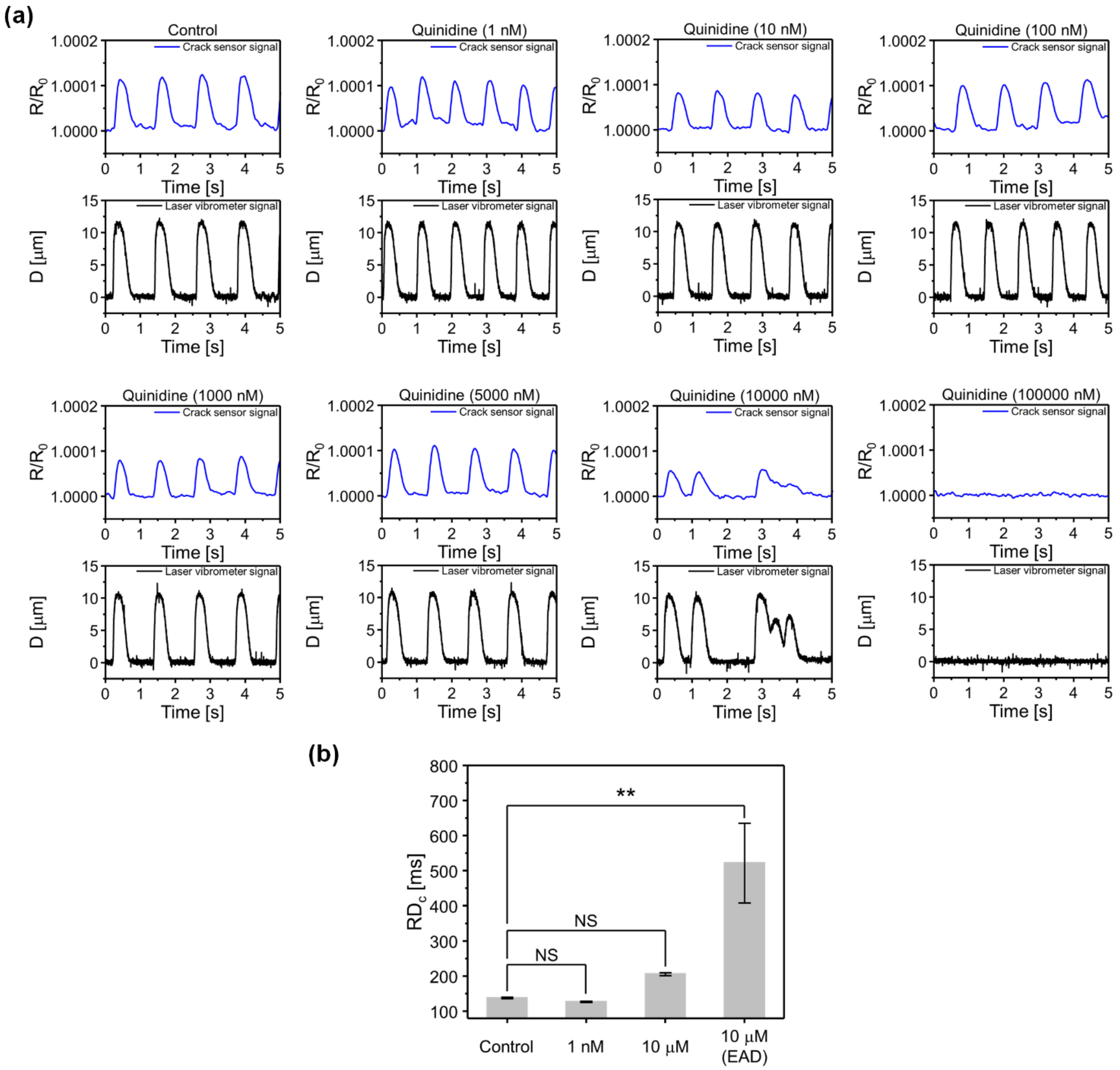

Supplementary Figure. 21. Real-time traces are representing the contractility of the cultured cardiomyocytes. **a**, Experimental results of cardiac contractile force and beating frequency according to Quinidine concentration. **b**, The rise and decay time (RD) for contractility analysis of the cultured cardiomyocytes were beat rate corrected (RDc) with Fridericia's formula (RDc = RD/interspike interval$^{1/3}$). (Non-significant (NS) and ** $p < 0.01$ measures by one-way ANOVA followed by Tukey's honest significant difference test.). Error bars are mean ± s.d. (n=5 biologically independent samples). D is representing displacement.

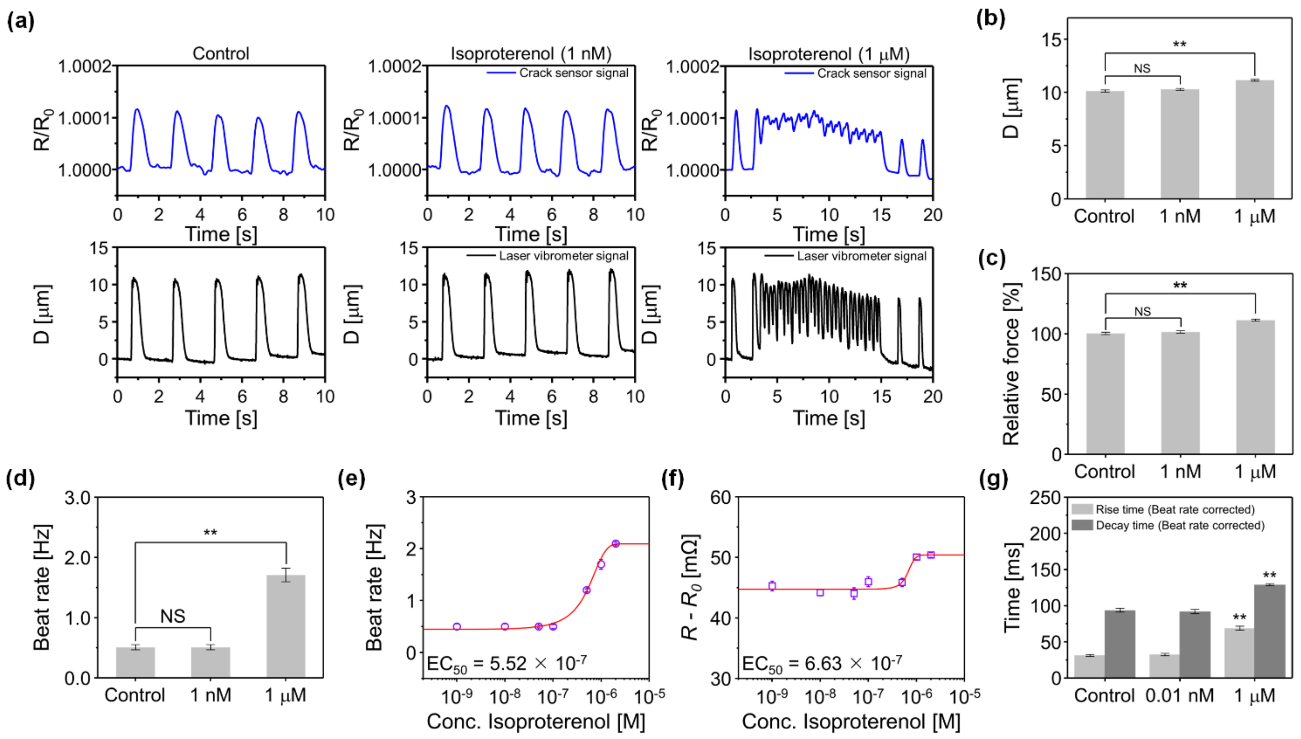

Supplementary Figure. 22. Effect of Isoproterenol on the cultured cardiomyocytes. **a**, Representative real-time traces of change in sensor resistance ratio and cantilever displacement owing to the contraction and relaxation of different concentrations of Isoprotereno treated cardiomyocytes. **b**, **c**, Bar plot depicting the cantilever displacement and relative contraction force generated by cardiomyocytes (** $p < 0.01$ measures by one-way ANOVA followed by Tukey's honest significant difference test.). **d**, Beat rate of cardiomyocytes at Isoproterenol concentrations of 1 nM and 1 μM. **e**, Beat rate of Isoproterenol treated cardiomyocytes at different concentrations (1 nM to 2 μM). **f**, Change in resistance of the cardiomyocytes seeded cantilever at different Isoproterenol concentrations (1 nM to 2 μM). **g**, Rise time and decay time of cardiomyocytes at Isoproterenol concentrations of 1 nM and 1 μM. The rise time and decay time were analyzed and beat rate corrected with Fridericia's formula (Rise time corrected (Rc) = R/interspike interval$^{1/3}$) and (Decay time corrected (Dc) = D/interspike interval$^{1/3}$), respectively. (** $p < 0.01$ measures by one-way ANOVA followed by Tukey's honest significant difference test.). Error bars are mean ± s.d. (n=5 biologically independent samples). NS and D are representing isplacement and non-significant, respectively.

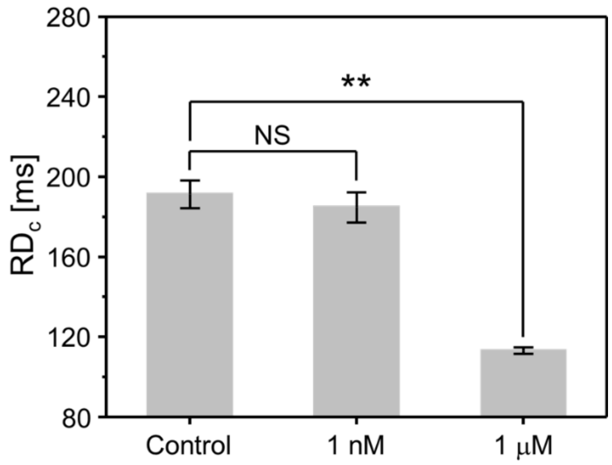

Supplementary Figure. 23. Rise and decay time of cardiomyocytes at Isoproterenol concentrations of 1 nM and 1 μM. The rise time and decay time were analyzed and beat rate corrected with Fridericia's formula (Rise time corrected (Rc) = R/interspike interval$^{1/3}$) and (Decay time corrected (Dc) = D/interspike interval$^{1/3}$), respectively. (** $p < 0.01$ measures by one-way ANOVA followed by Tukey's honest significant difference test.). Error bars are mean ± s.d. (n=5 biologically independent samples). NS is representing non-significant.

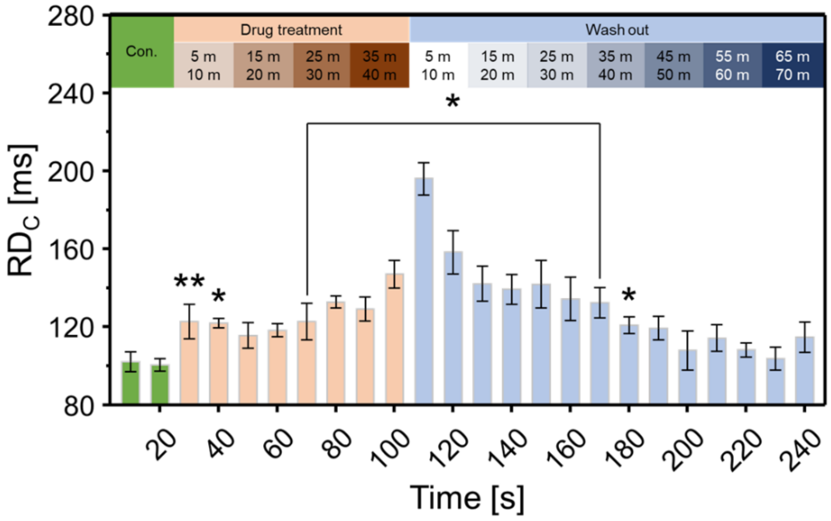

Supplementary Figure. 24. Rise and decay time of the 1 μM Verapamil treated cardiomyocytes at different drug treatment time. The rise and decay time (RD) for contractility analysis of the cultured cardiomyocytes were beat rate corrected (RDc) with Fridericia's formula (RDc = RD/interspike interval$^{1/3}$). (* $P < 0.05$, ** $P < 0.01$ measures by one-way ANOVA followed by Tukey's honest significant difference test. Error bars are mean ± s.d.). (n=5 biologically independent samples).

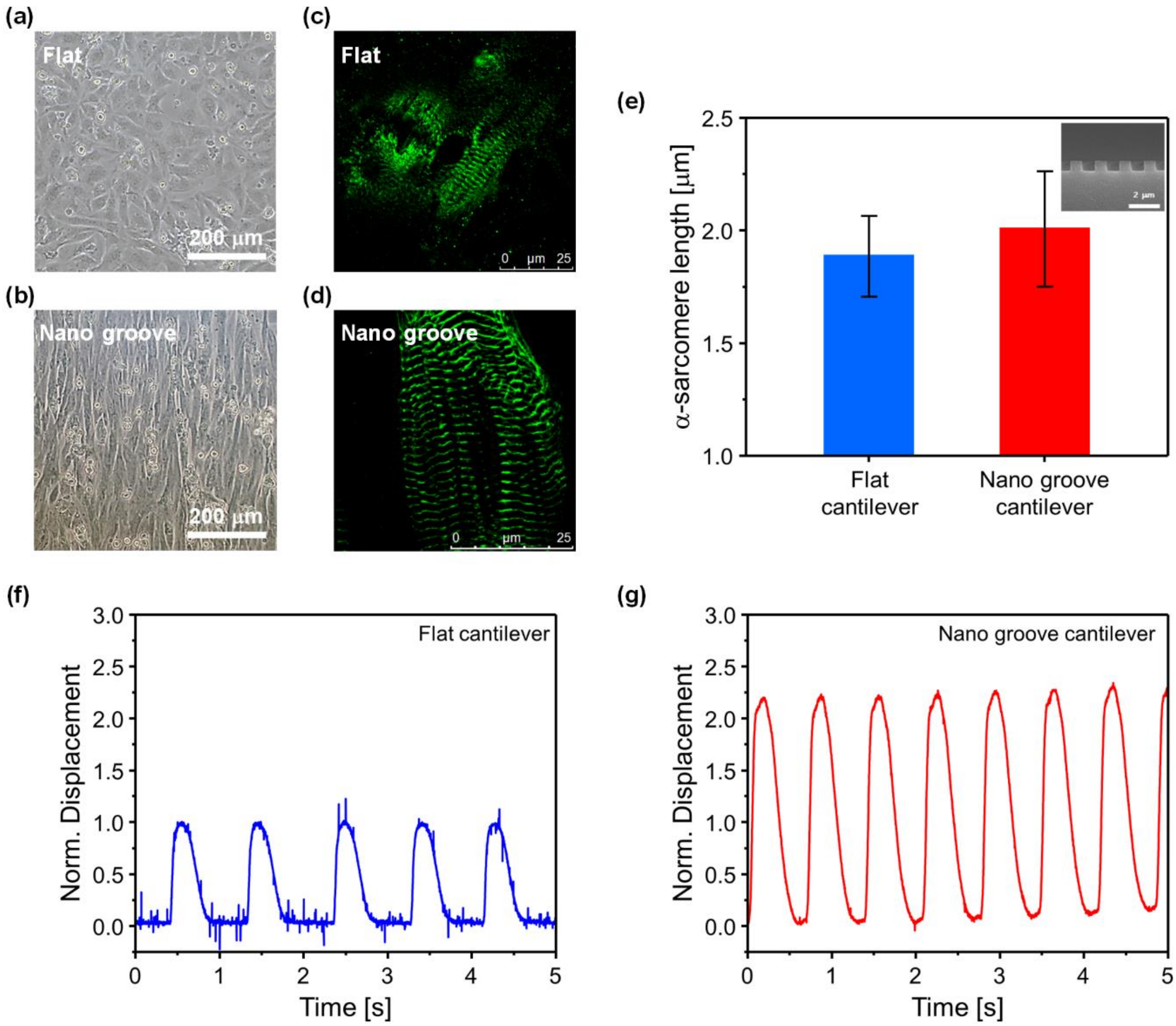

Supplementary Figure. 25. Effect of topographies on the growth of cardiomyocyte morphology. Optical microscope image of **a**, flat silicone rubber cantilever, **b**, nano-groove-patterned silicone rubber cantilever, **c**, flat silicone rubber cantilever (α-actinin), **d**, nano-groove-patterned silicone rubber cantilever (α-actinin). **e**, Sarcomere length analysis of cardiomyocytes at 7 days after culturing on different topographies. Normalized displacement for **f**, flat, and **g**, nano-grooved silicone rubber cantilevers. Error bars are mean ± s.d. (n=5 biologically independent samples).

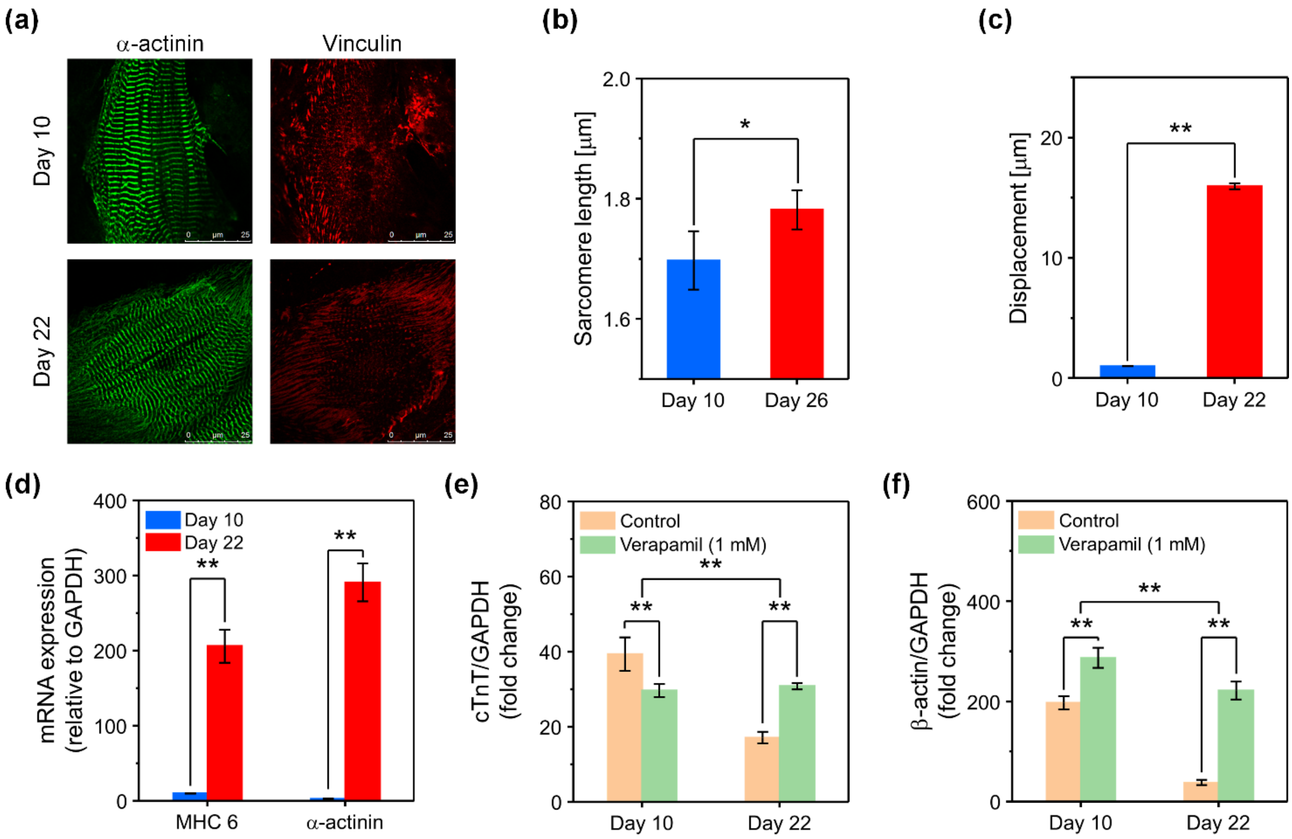

Supplementary Figure. 26. Immunofluorescence analysis of the cultured cardiomyocytes. **a**, Maturation of the cardiomyocytes investigated on day 10 and 22 of the culture day. Green: α-actinin, Red: Vinculin. **b**, Sarcomere length of cardiomyocytes on day 10 and 22 of culture day. Error bars are mean ± s.d., (n = 10)., * $p < 0.05$. **c**, Normalized displacement of the cantilever owing to the contraction force of cardiomyocytes on day 10 and 22 of the culture day., ** $p < 0.01$. **d**, mRNA expression (MHC 6, α-actinin) in cardiomyocytes on day 10 and 22 of the culture period., ** $p < 0.01$. **e**, Change in cTnT expression of the cardiomyocytes before and after drug treatment on day 10 and 22 of the culture period., ** $p < 0.01$ measures by two-way ANOVA followed by Tukey's honest significant difference test. **f**, Change in β-actin expression of the cardiomyocytes on day 10 and 22 of the culture day before and after drug treatment., ** $p < 0.01$ measures by two-way ANOVA followed by Tukey's honest significant difference test. Error bars are mean ± s.d. (n=5 biologically independent samples).

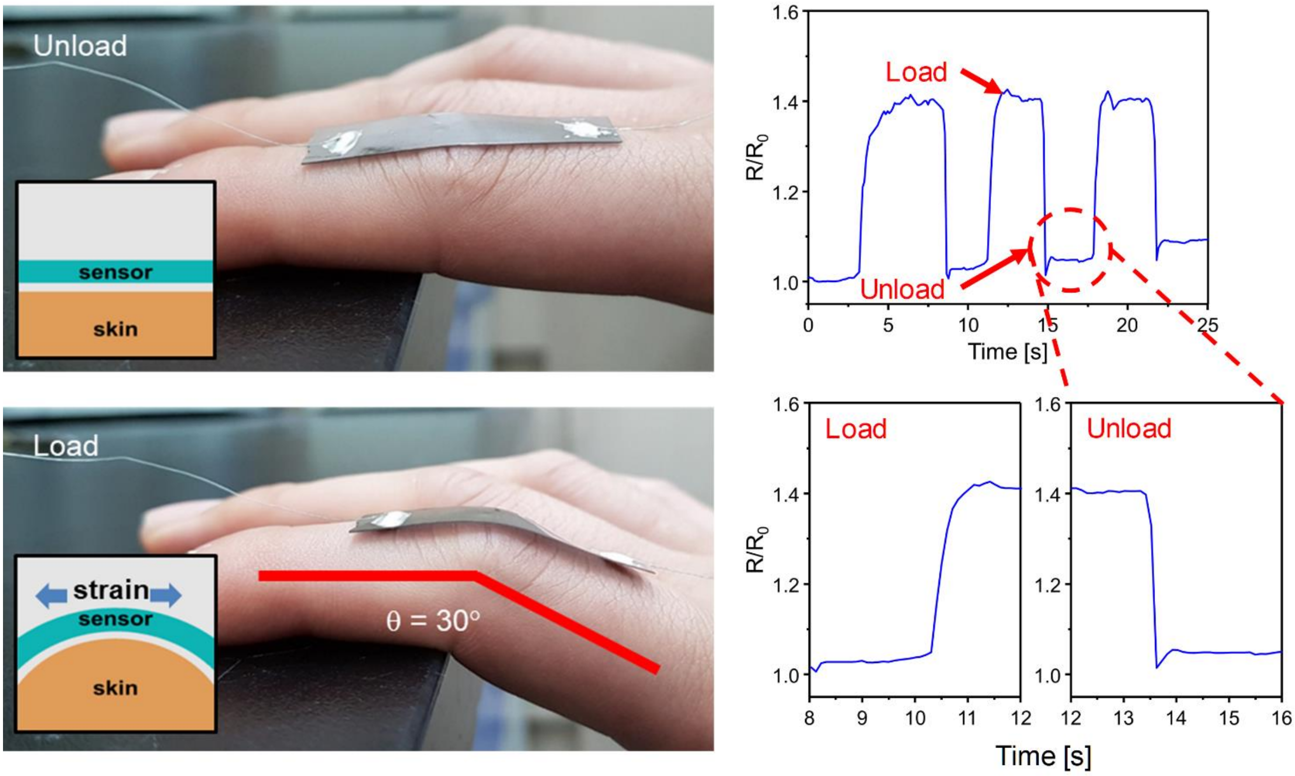

Supplementary Figure. 27. The flexibility of the proposed PDMS-encapsulated crack sensor. The photograph illustrates the unloading and loading of the crack sensor attached to the index finger. The graphs represent the change in the resistance ratio of the crack sensor according to the movement of index finger. Highly stable and flexible strain sensor based on PDMS-encapsulated metal cracks for wearable electronics applications.

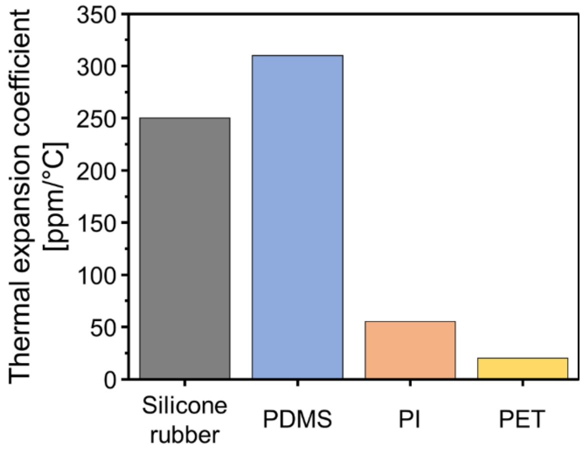

Supplementary Figure. 28. Mechanical properties of silicone rubber, PDMS, PI, and PET. The bar plot represents the thermal expansion coefficient of the silicone rubber, PDMS, PI, and PET.

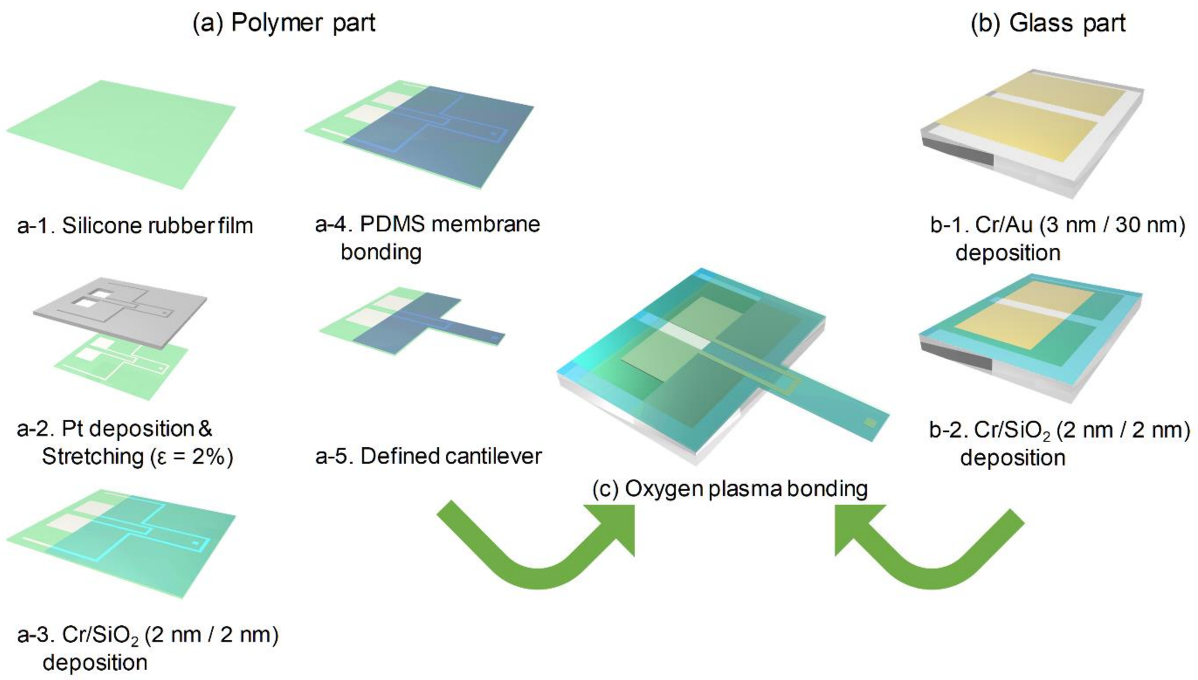

Supplementary Figure. 29. Schematic illustrating the fabrication process flow of the proposed sensor. **a-1**, Silicone rubber was placed on a PUA mold with nano-grooves. **a-2**, Deposition of a 20 nm Pt thin film on the silicone rubber and formation of cracks. **a-3**, Formation of an adhesion layer (Cr/SiO_2: 2 nm/2 nm) on the cracked Pt layer. **a-4**, Bonding of SiO_2 layer with PDMS via oxygen-based atmospheric plasma treatment. **a-5**, Defined the cantilever using a roll-to-plate and pinnacle die. **b-1**, Deposition of Cr/Au (3 nm/30 nm) using a thermal evaporation method. **b-2**, Cr/SiO_2 (2 nm/2 nm) was deposited using a thermal evaporator. C, The silicone rubber cantilever integrated with the crack sensor and the glass body were chemically bonded via oxygen-based atmospheric plasma treatment.

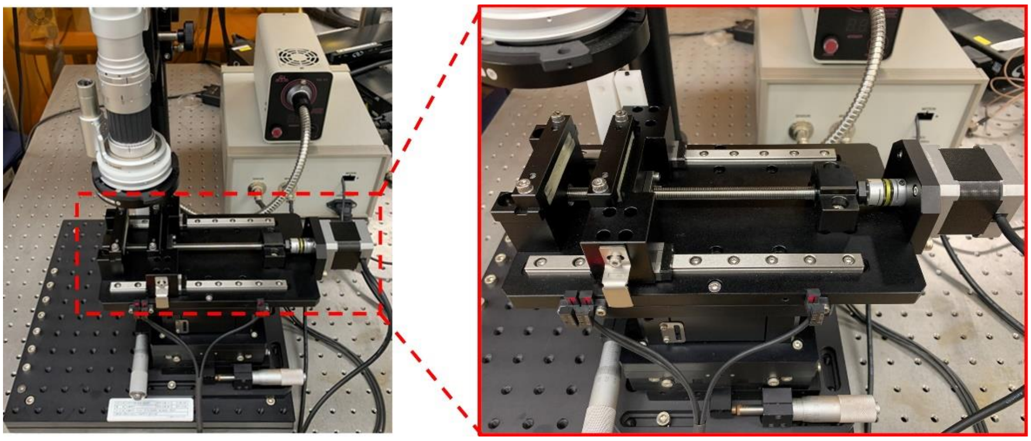

Supplementary Figure. 30. Photograph of the tensile testing machine used for the fabrication of crack sensors.

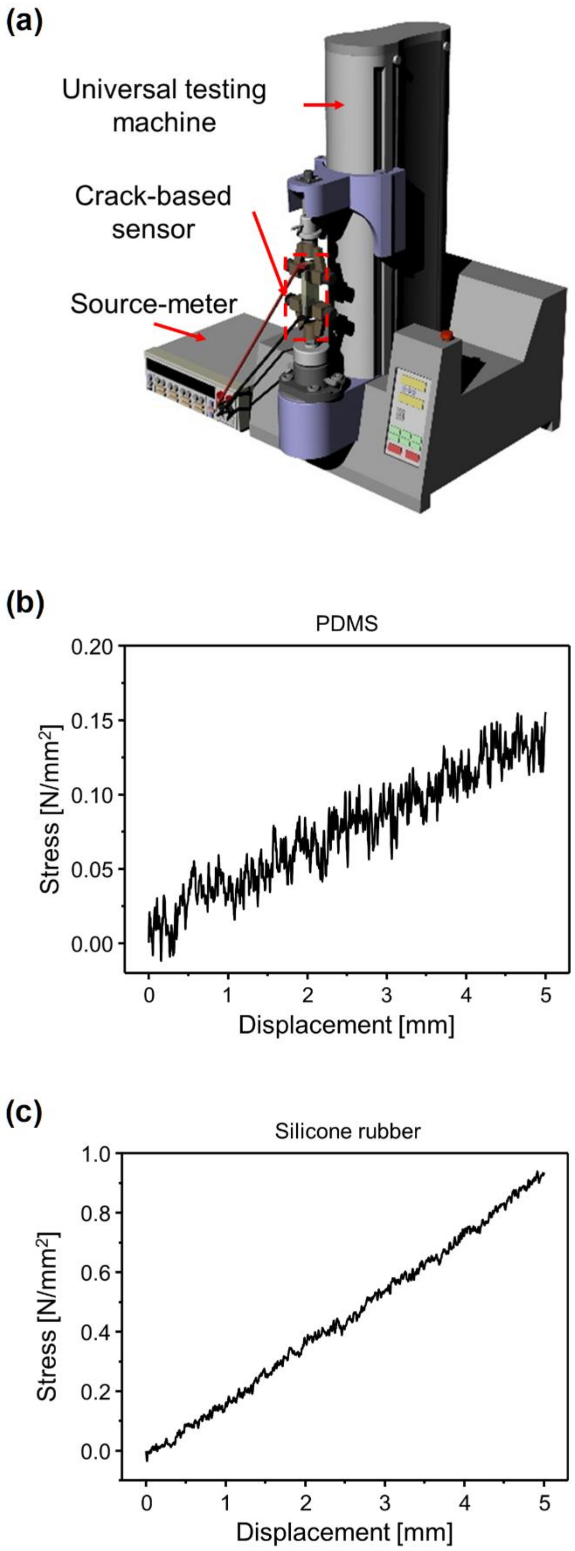

Supplementary Figure. 31. Mechanical properties of the PDMS and silicone rubber. **a**, Schematic illustrating the experimental setup used to measure the mechanical properties of the PDMS and silicone rubber. Stress-strain curves of the **b**, PDMS, and **c**, Silicone rubber as a function of displacement.

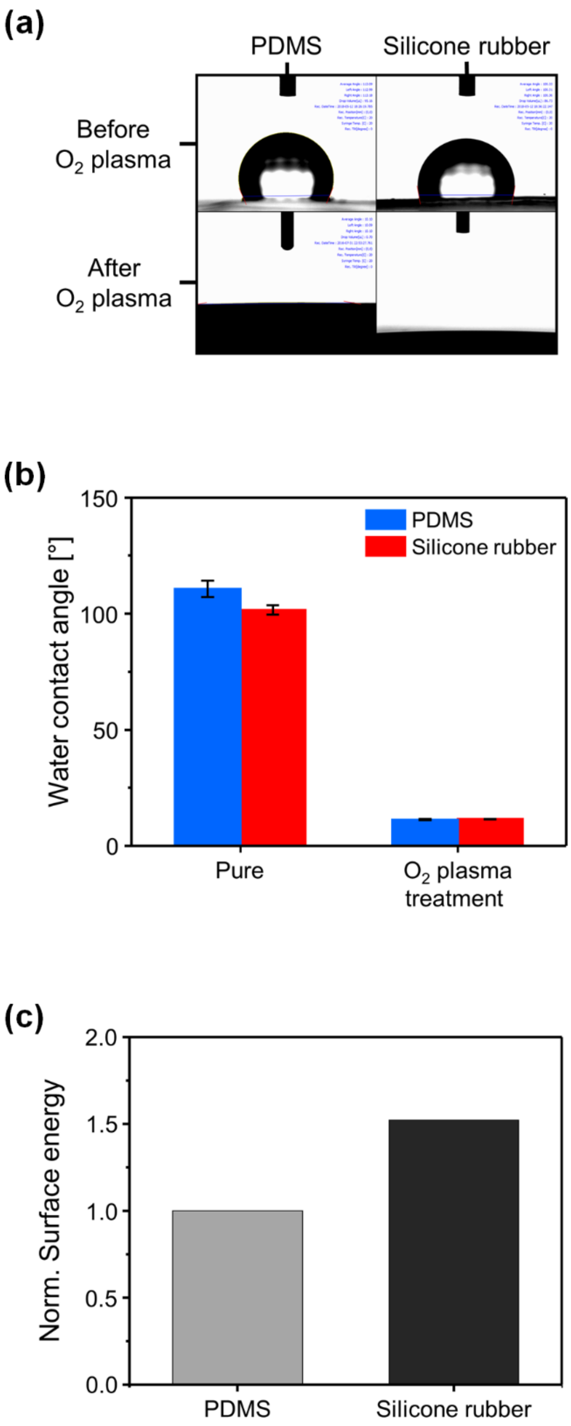

Supplementary Figure. 32. Surface modification of the cell culture substrate. **a**, The optical images show the water droplet on the PDMS and silicone rubber before and after the O_2 plasma treatment. **b**, Bar plot illustrating the water contact angle of the PDMS and silicone rubber before and after the O_2 plasma treatment. **c**, The surface energy of PDMS and silicone rubber before the O_2 plasma treatment. Error bars are mean ± s.d. (n=5).

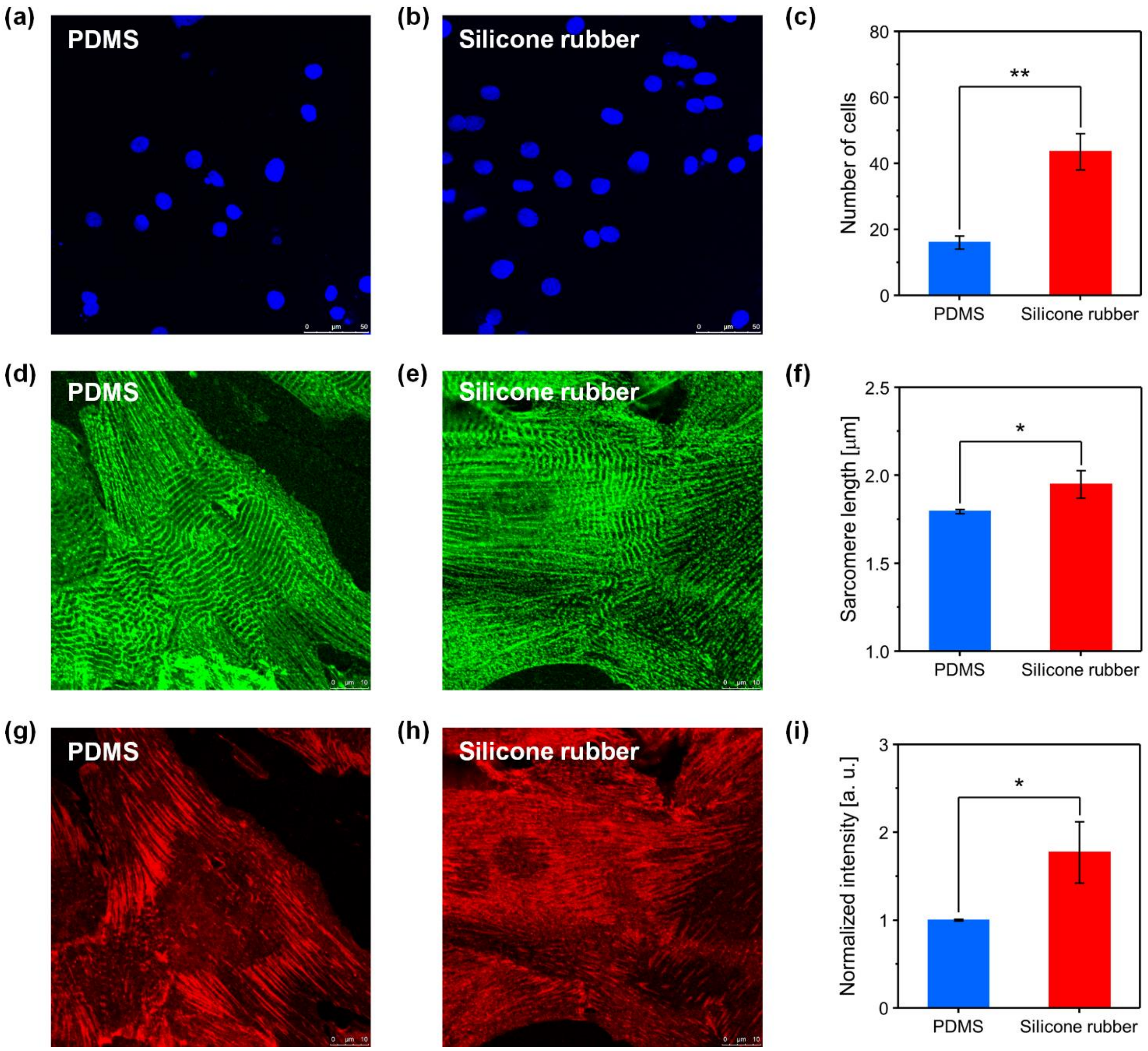

Supplementary Figure. 33. Immunocytochemistry staining images of cardiomyocytes cultured on PDMS and silicone rubber substrates. **a–b**, DAPI staining images of the cardiomyocytes cultured on PDMS and silicone rubber on day 7 of the culture period. **c**, The number of cells observed in PDMS and silicone rubber cantilever on day 7 of the culture period (area ~250 μm × 250 μm). **d–e**, α-actinin staining images of the cultured cardiomyocytes in PDMS and silicone rubber cantilever on day 7 of the culture period (area ~250 μm × 250 μm). **f**, Measured sarcomere length of the cardiomyocytes cultured on PDMS and silicone rubber substrates on 7th day of the culture period. **g–h**, Vinculin staining images of the cultured cardiomyocytes in PDMS and silicone rubber cantilever on day 7 of the culture period (area ~250 μm × 250 μm). **i**, Normalized vinculin intensity of the cardiomyocytes cultured on PDMS and silicone rubber substrates on 7th day of the culture period. Error bars are mean ± s.d. (n=10 biologically independent samples)., * $P < 0.05$, ** $P < 0.01$.

Supplementary Notes

Supplementary Note. 1. Supplementary Figure 1 describes the mechanism of crack sensors that are distinctly different from the commercial strain gage sensor. The cured silicone rubber cantilever integrated with the proposed crack sensor was chemically bonded to PDMS by plasma treatment in an oxygen atmosphere for long-term use in conductive culture medium. Since the metal layer can-not chemically bond with PDMS-encapsulation layer even after the plasma treatment, an adhesion layer of Cr/SiO_2 was deposited on the Pt layer for appropriate adhesion between the Pt metal layer and the PDMS layer. The proposed crack sensor retains its non-linear characteristics same as the conventional crack sensor even after embedding the Cr, SiO_2, and PDMS.

The physical model in Supplementary Figure 1 illustrates the mechanism of the proposed crack sensor. At the initial state, all the lips of the crack are in a closed state. When strain is applied to the crack sensor (State 1), all the lips are instantly opened, and the resistance increases as the electrical pathways are blocked. Then the lips are partially closed due to the compressive force of the Poisson's ratio (State 2) and partially open the electrical path. Thus, through the repetition of states 1 and 2, the crack sensor exhibits an exponential resistance increase rather than momentarily increase in resistance. Finally, when the applied strain approaching or exceeding 1% all the lips are opened, and the resistance reaches infinity (state 5). After withdrawing the applied tension, the gap between the cracks closed, and the initial resistance of the crack sensor is completely restored.

Detailed theoretical modeling and the non-linear characteristics of the crack sensor is explained in detail in the supporting information according to our previous study [1, 2].

For that sensor due to a technology of producing large unidirectional strain, the free cracks cut the sensor strip through so that the normalized conductance S of the sensor vs strain ε

$$S = \int_{\varepsilon}^{\infty} \mathbf{P}(x)dx \qquad (1)$$

was determined by the probability distribution function (pdf) $\mathbf{P}(x)$ of the steps on a crack lip[15] making contacts between the lips. For a free crack we found a Supplementary Equation for $\mathbf{P}(x)$ with the only "size" parameter – the strain ε_0 that corresponds to the crack gap width $k\,\varepsilon_0$ being about the grain size $x_0 = k\,\varepsilon_0$

$$\mathbf{P}(x) = \mathbf{P}(1/x)/x^2, \qquad (2)$$

where $x = \frac{\varepsilon}{\varepsilon_0}$ and k is the proportionality factor to be defined by relating the crack gap width to the

strain[15]. k can be different for the different material realization of parallel crack systems and should be obtained from experiment. Physically, Supplementary Equation (2) states that tiny steps of the crack asperity made by the shifts of grains are distributed the same as the large steps made by grain piling because the substrate elastic field being scale-less and thus having no characteristic length may not distinguish between tiny and large meandering asperity. Among solutions of Supplementary Equation (2), one may choose either the *log-normal* pdf

$$\mathbf{P}(\varepsilon) = \frac{1}{\varepsilon\mu\sqrt{\pi}}\exp\left(-\frac{(\ln(\varepsilon/\varepsilon_0))^2}{\mu^2}\right) \quad (3)$$

or a nearly identical *log-logistic* pdf

$$\mathbf{P}(\varepsilon) = \frac{B}{\varepsilon_0}\frac{(\varepsilon/\varepsilon_0)^{B-1}}{(1+(\varepsilon/\varepsilon_0)^B)^2} \quad (4),$$

where μ and B are parameters of the pdf.

Both distributions of Supplementary Equations (3) and (4) belong to the class of so-called skew distributions with long tails. As we discussed in Ref. 1, the non-zero probability of large but rare contacts between crack lips lies in the essence of the mechanism of the conduction through the crack and is, therefore, in concordance with the tailed distributions. With Supplementary Equation (3), Supplementary Equation (1) gives for the resistance $R = 1/S$ as a function of strain the following:

$$R = 2/\left(1 - \mathrm{erf}\left(\frac{\ln\left(\frac{\varepsilon}{\varepsilon_0}\right)}{\mu}\right)\right), \quad (5)$$

$\mathrm{erf}(x)$ is the error function. Supplementary Equation (5) renders the normalized resistance that remarkably fits the experiment[15] for the strains up to 2%. At the same time, one can show that the log-logistic pdf of Supplementary Equation (4) together with Supplementary Equation (1) leads to

$$R = 1 + (\varepsilon/\varepsilon_0)^B \quad (6)$$

that fits the experiment[15] with fitting parameters $\varepsilon_0 = 0.39$ and $B = 2.39$ with the same accuracy as the log-normal pdf of Supplementary Equation (5). Yet, the *power-law* function of Equation (6) is much simpler than the error function in the Supplementary Equation (5). We may suggest this universal power law for data fitting by experimentalists who study free parallel cracks.

The applied external strain opened the lips and reduces the electrical paths of the crack sensor. The resistance component generated at the contact point of the cracks and the gap between the cracks behaves like resistance and capacitance parallel circuit (Supplementary Figure 1b).

The impedance component of the crack sensor shown in the equivalent circuit model (Supplementary Figure 1b) of the PDMS-encapsulated crack sensor integrated silicone rubber cantilever can be represented as a Supplementary Equation (7). However, we have carried out all the sensing performance measurement of the crack sensor through DC power; hence, the change in capacitance (acts as open circuits in a DC condition) due to the applied tensile strain is not considered in this present work. Therefore, the equivalent circuit of the crack sensor can be expressed, as shown in Supplementary Figure 1c.

$$\boldsymbol{Z} = \frac{\boldsymbol{R}}{\boldsymbol{1+j\omega RC}} \quad , \quad \boldsymbol{\omega} = 2\pi f \quad , \qquad (7)$$

(Supplementary Figure 1)

Supplementary Note. 2. Preliminary characteristics of the sensor. The cracks formed on thin metal films have a shape in which stress is concentrated at the crack ends. When the gap distances of the cracks are increased, the electrical resistance is increased dramatically owing to the decrease of the tunneling current as well as the number of the electrical contacts of each crack. Optical microscope images were used to monitor the number of contacts of each crack in the fabricated crack sensor. Supplementary Figure 2a shows an optical microscope image depicting an increase in cracks due to tensile strain, also evidenced by the intensity changes, as shown in Supplementary Figure 2b. An increase in tensile strain in the range of 0–2% was found to proportionally increase the number of cracks. Tensile testing equipment (3342 UTM, Instron Co., Norwood, MA, USA) was employed to measure the electrical conductivity changes of the fabricated crack sensor according to tensile strain changes in the range of 0–0.3% and 0–1%, respectively. The resistance changes on the fabricated crack sensor were measured in real-time using a Lab-VIEW-based data acquisition system (PXI-4071, National Instruments Inc., Austin, TX, USA). The standard deviation of the measured value was 0.01 Ω. As shown in Supplementary Figure 2(c, d), the electrical resistance of the crack sensor increased 1.7 times for a 0.3% strain and increased approximately 9,000 times for a 1% strain, respectively. The crack sensors used in this test included two types with and without PDMS-encapsulation layer. It was confirmed to have the same sensitivity in air regardless of the presence of the encapsulation layer. The results of the hysteresis of the crack sensor are shown in Supplementary Figure 2e. Owing to the inherent characteristics of the polymer material, there was a certain hysteresis value, but it was significantly reduced compared with that of the PDMS cantilever. The PDMS-

encapsulated crack sensor had a GF of 9×10^6 at a 1% strain. The hysteresis of the PDMS-encapsulated crack sensor integrated silicone rubber cantilever in the tensile range of 0-0.5% is shown in Supplementary Figure 3. The obtained experimental data (Supplementary Figure 3) demonstrated that the hysteresis does not appear at low tensile range where the crack sensor-operated to measure cell contraction force of the cardiomyocytes. In addition, the response time of the crack sensor is very similar to that of the conventional optical (laser vibrometer) method (Supplementary Figure 2 and Supplementary Figure 3).

Supplementary Note. 3. As for as the hysteresis concerns, the proposed sensor exhibits the hysteresis only at the higher strain range. At the lower tensile in the range of 0-0.5%, the crack sensor shows no hysteresis (Supplementary Figure 3). In this present investigation, the maximum strain produced by the cultured cardiomyocytes is ~0.03%, which causes ~100 μm cantilever displacement (Supplementary Figure 3).

Supplementary Note. 4. Further, we have carried out the additional simulation and experimental analysis to show the effect of the thermal coefficient on the cantilever displacement. The temperature-dependent deformation of the cantilever composed of the silicone rubber cantilever, silicone rubber and PDMS (Silicone rubber + PDMS) was analyzed using the commercial finite element analysis program (ANSYS). As a result of the thermal expansion analysis, the deflection of the cantilever increases as the temperature increases (Supplementary Figure 4). The temperature-dependent displacement of the cantilever composed of silicone rubber and PDMS was measured using a laser displacement sensor under a home-made stage-top incubator environment (Supplementary Figure 4).

Supplementary Note. 5. The bar plot shows the displacement of the cantilever as a function of temperature. The deformation of the cantilever was found to be increased with increasing the incubator temperature. At 40 °C the simulation studies show the displacement of ~106 μm, whereas the experimental analysis shows the displacement of ~66 μm. However, the cantilever showed no further deformation once the temperature stabilized inside the incubator. Supplementary Figure 5b shows the PDMS-encapsulated silicone rubber cantilever deformation at 40 °C as a function of time. The time-dependent studies showed almost stable cantilever deformation and remain constant indicating the reliability of the proposed device (Supplementary Figure 5).

Supplementary Note. 6. The effect of temperature on the deformation of the cantilever is not significant owing to the use of low thermal conductivity polymer materials. Supplementary Figure 6a shows the change in resistance of the PDMS-encapsulated crack sensor integrated silicone rubber cantilever as a function of time at different incubator set temperature. As observed, the resistance of the sensor increases with increasing temperature and stabilized approximately 10 min and maintained stable resistance with further increasing time. Supplementary Figure 6b shows the displacement of the PDMS-encapsulated crack sensor integrated silicone rubber cantilever sensor at 40 °C. The sensor showed almost stable displacement over 20 min indicating the negligible thermal effect on the proposed sensor (Supplementary Figure 6).

Supplementary Note. 7. The noise level of the crack sensor shows a high noise compared to the optical measuring method using the commercial laser displacement meter. However, as mentioned on page 3 of the text, the optical measurement method using the laser displacement sensor requires a motorized stage for the movement of the laser displacement sensor or multiple laser displacement sensors for the high-throughput screening, it is difficult to analyze various drugs in real-time. High-throughput drug screening is possible by employing the PDMS-encapsulated crack sensor integrated silicone rubber cantilever. Also, the proposed crack sensor has a 583 times higher signal-to-noise ratio (SNR) than the conventional piezo-resistive sensor, allowing accurate analysis of changes in the cardiomyocyte contractile force (Supplementary Figure 8). In addition, the proposed crack sensor has a phenomenon that the baseline increases or decreases according to the fluctuation of the internal temperature of the stage-top incubator due to temperature-dependent characteristics. This problem can be kept constant by using the active-dummy method used in commercial strain gauges (Supplementary Figure 8).

Supplementary Note. 8. The proposed crack sensor exhibits high sensitivity and non-linear characteristics. Owing to the non-linear characteristics, the crack sensor shows the considerable resistance ratio variation (R / R_0) ~ 90,000 at higher strain range (0-1%) and relatively small variation of ~1.7 at the lower strain range (0-0.3%). However, the strain produced by the cultured cardiomyocytes on the cantilever is minimal. In this present study, the maximum strain produced by the cultured cardiomyocytes on the cantilever is ~0.03%, which causes ~100 μm cantilever displacement. In addition, the gauge factor of the crack sensor is ~156 times higher than the commercial piezoresistive sensor at 0.03% strain (Supplementary Figure 9). Therefore, the crack sensor is highly sensitive enough to detect even the smaller variation in the displacement of the cantilever caused by the contractile force of the cultured

cardiomyocytes (Supplementary Figure 9).

Supplementary Note. 9. In addition, the response time of the crack sensor is very similar to that of the conventional optical (laser vibrometer) method. Therefore, it is easy to analyze the contraction force of the cardiomyocytes changing in real-time. A 100 μm displacement was applied to the integrated silicone rubber cantilever using a motorized stage and measured the displacement using a laser vibrometer and crack sensor. The laser vibrometer and crack sensor showed a response time of 121 and 123 ms and remained constant output after the displacement was applied (Supplementary Figure 10). It is confirmed that this method can be used to analyze cardiomyocyte contractility and heart rate (Supplementary Figure 10).

Supplementary Note. 10. Further, the reproducibility of the crack sensor has been verified by fabricating the five crack sensors under similar manufacturing conditions. Supplementary Figure 11 shows the change in resistance of the fabricated crack sensors at different applied strain ranging from 0 to 0.7 %. As shown in the figure the sensors fabricated under similar manufacturing conditions showed comparatively uniform characteristics with the maximum change in resistance ratio of 44.17± 3.67, indicating the high-performance reliability of the proposed crack sensors (Supplementary Figure 11).

Supplementary Note. 11. We have fabricated the crack sensor arrays by integrating three cantilevers in a glass body and analyzed the characteristics of each cantilever by applying a 0.03% strain. The initial resistance of the fabricated cantilever array was found to be ~276 ± 8.2 Ω, and the resistance increased to ~288 ± 8.1 Ω after applying 0.3% of strain. As shown in Supplementary Figure 12, the fabricated sensors showed an almost similar change in resistance trend with the maximum resistance ratio (R/R_0) of 1.0437 ± 0.002, indicating the high reliable performance of the proposed crack sensors (Supplementary Figure 12).

Supplementary Note. 12. The resistance ratio of the sensors was found to be almost similar. The smaller variation in the resistance variation trend between the three sensors could be attributed to the different behavior seeded cardiomyocytes under the cell culture medium. The obtained results demonstrating the high reliability and repeatability of the proposed PDMS-encapsulated crack sensor integrated with silicone

rubber cantilever (Supplementary Figure 18).

Supplementary Note. 13. Immunocytochemistry staining analysis. To demonstrate the improved performance of the proposed sensing platform, we have performed the traditional gene expression and protein localization analysis. Supplementary Figure 23 shows the immunofluorescence of cardiomyocytes obtained from the PDMS-encapsulated crack sensor integrated silicone rubber cantilever on day 10 and 22 of the culture period. The cardiomyocytes obtained from day 22 are found to be more elongated in the direction of grooves and formed an aligned cell tissue like that seen in vivo compared to that of cardiomyocytes obtained from day 10 (Supplementary Figure 23a). The sarcomere length of cardiomyocytes on day 22 was found to be 1.78 ± 0.032 μm, whereas, the sarcomere length of cardiomyocytes on day 10 was 1.69 ± 0.048 μm (Supplementary Figure 23b). The displacement of the cantilever on day 10 and 22 were 1.01 ± 0.021 μm and 15.97 ± 0.232 μm, respectively (Supplementary Figure 23c). RT-qPCR of the four genes analysis confirms the improved contractile force (MHC 6, cTnT), higher maturation (α-actinin, β-actin), and enhanced contractility of cardiomyocytes on day 22 compared to that of cardiomyocytes on day 10. The MHC 6 and α-actinin of cardiomyocytes on day 10 and 22 were 10 ± 0.277 and 206 ± 21.816, respectively (Supplementary Figure 23d). The cTnT expression of cardiomyocytes on day 10 was 39.4 ± 4.464 at the control state, and it decreased to 29.7 ± 1.804 after treating cardiomyocytes with 1 μM Verapamil. Whereas, the cTnT expression of the cardiomyocytes on day 22 was found to be increased to 30.8 ± 0.865 after μM Verapamil treatment compared to 17.1 ± 1.545 at the control state (Supplementary Figure 23e). The β-actin of the cardiomyocytes on day 10 and day 22 of the culture period before and after 1 μM Verapamil were 197 ± 13.373, 286.79 ± 20.169 and 38 ± 5.131, 221.91 ± 17.581, respectively (Supplementary Figure 23e) (Supplementary Figure 26).

Supplementary References

[1] Kang, D. et al. Ultrasensitive mechanical crack-based sensor inspired by the spider sensory system. *Nature*. **516**, 222 (2014).

[2] Choi, Y. W. et al. Ultra-sensitive Pressure sensor based on guided straight mechanical cracks. *Sci Rep*. **7**, 40116 (2017).
