# Supplementary material for: Highly durable crack sensor integrated with silicone rubber cantilever for measuring cardiac contractility
Source: Nat Commun. 2020 Jan 27;11:535. doi: 10.1038/s41467-019-14019-y (PMC6985253; doi:10.1038/s41467-019-14019-y)
Supplement: Supplementary file 3 — Reporting Summary [file 41467_2019_14019_MOESM3_ESM.pdf]

nature research

Corresponding author(s): Prof. Dong-Weon Lee

Last updated by author(s): Nov 14, 2019

Reporting Summary

Nature Research wishes to improve the reproducibility of the work that we publish. This form provides structure for consistency and transparency in reporting. For further information on Nature Research policies, see Authors & Referees and the Editorial Policy Checklist .

Statistics

For all statistical analyses, confirm that the following items are present in the figure legend, table legend, main text, or Methods section.

n/a	Confirmed	
☐	☒	The exact sample size (*n*) for each experimental group/condition, given as a discrete number and unit of measurement
☐	☒	A statement on whether measurements were taken from distinct samples or whether the same sample was measured repeatedly
☐	☒	The statistical test(s) used AND whether they are one- or two-sided<br>*Only common tests should be described solely by name; describe more complex techniques in the Methods section.*
☐	☒	A description of all covariates tested
☐	☒	A description of any assumptions or corrections, such as tests of normality and adjustment for multiple comparisons
☐	☒	A full description of the statistical parameters including central tendency (e.g. means) or other basic estimates (e.g. regression coefficient) AND variation (e.g. standard deviation) or associated estimates of uncertainty (e.g. confidence intervals)
☐	☒	For null hypothesis testing, the test statistic (e.g. *F*, *t*, *r*) with confidence intervals, effect sizes, degrees of freedom and *P* value noted<br>*Give P values as exact values whenever suitable.*
☐	☒	For Bayesian analysis, information on the choice of priors and Markov chain Monte Carlo settings
☐	☒	For hierarchical and complex designs, identification of the appropriate level for tests and full reporting of outcomes
☐	☒	Estimates of effect sizes (e.g. Cohen's *d*, Pearson's *r*), indicating how they were calculated

Our web collection on statistics for biologists contains articles on many of the points above.

Software and code

Policy information about availability of computer code

Data collection	LabView 2009, LAS AF 2.63
Data analysis	OriginPro 2018 SR1, MATLAB R2018a, ImageJ 1.52a, SarcOptiM-High Frequency Online Sarcomere Length Measurement , GraphPad Prism 7.04

For manuscripts utilizing custom algorithms or software that are central to the research but not yet described in published literature, software must be made available to editors/reviewers. We strongly encourage code deposition in a community repository (e.g. GitHub). See the Nature Research guidelines for submitting code & software for further information.

Data

Policy information about availability of data

All manuscripts must include a data availability statement. This statement should provide the following information, where applicable:

- Accession codes, unique identifiers, or web links for publicly available datasets
- A list of figures that have associated raw data
- A description of any restrictions on data availability

All relevant data supporting the findings of this study are available herein and in the Supplementary Information files, or from the corresponding author upon reasonable request. "The data underlying Figures 2c,f,i, 3c-e, 4c-f, 5, 6b-g, 7b-g, and 8, as well as Supplementary Figures 2b, 5a, 8, 9, 11, 16, 19b, 22b-g, 25e, 26b-f, 28, 32b-c, and 33c,f,i are provided as a Source Data file."

Field-specific reporting

Please select the one below that is the best fit for your research. If you are not sure, read the appropriate sections before making your selection.

- [x] Life sciences
- [ ] Behavioural & social sciences
- [ ] Ecological, evolutionary & environmental sciences

For a reference copy of the document with all sections, see nature.com/documents/nr-reporting-summary-flat.pdf

Life sciences study design

All studies must disclose on these points even when the disclosure is negative.

Sample size	No sample-size calculations were performed. Sample size was determined to be adequate based on the magnitude and consistency of measurable differences between groups.
Data exclusions	On principle, data were only excluded for failed experiments, reasons for which included suboptimal activation and microbial contamination.
Replication	All experiments were repeated at least three times. Data presented in this study is representative for the different repeated measurement that combined in one final figure.
Randomization	Randomization of Sprague-Dawley rat. The heart from a Sprague-Dawley rats used in the experiment are within day 1 to 3.
Blinding	No blinding was performed for these studies.

Reporting for specific materials, systems and methods

We require information from authors about some types of materials, experimental systems and methods used in many studies. Here, indicate whether each material, system or method listed is relevant to your study. If you are not sure if a list item applies to your research, read the appropriate section before selecting a response.

Materials & experimental systems

n/a	Involved in the study	
[ ]	[x]	Antibodies
[x]	[ ]	Eukaryotic cell lines
[x]	[ ]	Palaeontology
[ ]	[x]	Animals and other organisms
[x]	[ ]	Human research participants
[x]	[ ]	Clinical data

Methods

n/a	Involved in the study	
[x]	[ ]	ChIP-seq
[x]	[ ]	Flow cytometry
[x]	[ ]	MRI-based neuroimaging

Antibodies

Antibodies used	1. Vinculin- Sigma Aldrich, V9131, Mouse, IgG1.<br>2. α-actinin- Abcam, ab137346, Rabbit, IgG.<br>3. DAPI- Thermo Fisher Scientific, Invitrogen, Prolong Gold Antifade Mountant wIth DAPI, P36931
Validation	All antibodies used in this manuscript have been validated for this specific use by the respective vendor.

Animals and other organisms

Policy information about studies involving animals; ARRIVE guidelines recommended for reporting animal research

Laboratory animals	NRVM (Neonatal Rat Ventricular Myocytes) isolated from the heart from a Sprague-Dawley rat between days 1 and 3, without distinction of sex.
Wild animals	The study did not involve wild animals.
Field-collected samples	The study did not involve samples collected from the field.
Ethics oversight	All animal experiments were performed in accordance with protocols approved by the protocols approved by the Animal Ethics Committee at Chonnam National University-Republic of Korea.

Note that full information on the approval of the study protocol must also be provided in the manuscript.
